# Microbial community regulation and performance enhancement in gas biofilters by interrupting bacterial communication

**DOI:** 10.1186/s40168-022-01345-5

**Published:** 2022-09-19

**Authors:** Yong-Chao Wang, Yu-Ting Lin, Can Wang, Zhen Tong, Xu-Rui Hu, Ya-Hui Lv, Guan-Yu Jiang, Meng-Fei Han, Ji-Guang Deng, Hsing-Cheng Hsi, Chung-Hak Lee

**Affiliations:** 1 School of Environmental Science and Engineering, Tianjin University, Tianjin, 300072 China; 2 Tianjin Key Lab of Indoor Air Environmental Quality Control, Tianjin, 300072 China; 3 College of Environmental and Energy Engineering, Beijing University of Technology, Beijing, 100124 China; 4 Graduate Institute of Environmental Engineering, National Taiwan University, No. 1, Sec. 4, Roosevelt Rd, Taipei, 106 Taiwan; 5 School of Chemical and Biological Engineering, Seoul National University, Seoul, 08826 Republic of Korea

**Keywords:** Bacterial communication, Microbial community regulation, Quorum quenching, Function genes, Biofilter

## Abstract

**Background:**

Controlling excess biomass accumulation and clogging is important for maintaining the performance of gas biofilters and reducing energy consumption. Interruption of bacterial communication (quorum quenching) can modulate gene expression and alter biofilm properties. However, whether the problem of excess biomass accumulation in gas biofilters can be addressed by interrupting bacterial communication remains unknown.

**Results:**

In this study, parallel laboratory-scale gas biofilters were operated with *Rhodococcus* sp. BH4 (QQBF) and without *Rhodococcus* sp. BH4 (BF) to explore the effects of quorum quenching (QQ) bacteria on biomass accumulation and clogging. QQBF showed lower biomass accumulation (109 kg/m^3^) and superior operational stability (85–96%) than BF (170 kg/m^3^; 63–92%) at the end of the operation. Compared to BF, the QQBF biofilm had lower adhesion strength and decreased extracellular polymeric substance production, leading to easier detachment of biomass from filler surface into the leachate. Meanwhile, the relative abundance of quorum sensing (QS)-related species was found to decrease from 67 (BF) to 56% (QQBF). The QS function genes were also found a lower relative abundance in QQBF, compared with BF. Moreover, although both biofilters presented aromatic compounds removal performance, the keystone species in QQBF played an important role in maintaining biofilm stability, while the keystone species in BF exhibited great potential for biofilm formation. Finally, the possible influencing mechanism of *Rhodococcus* sp. BH4 on biofilm adhesion was demonstrated. Overall, the results of this study achieved excess biomass control while maintaining stable biofiltration performance (without interrupting operation) and greatly promoted the use of QQ technology in bioreactors.

**Graphical Abstract:**

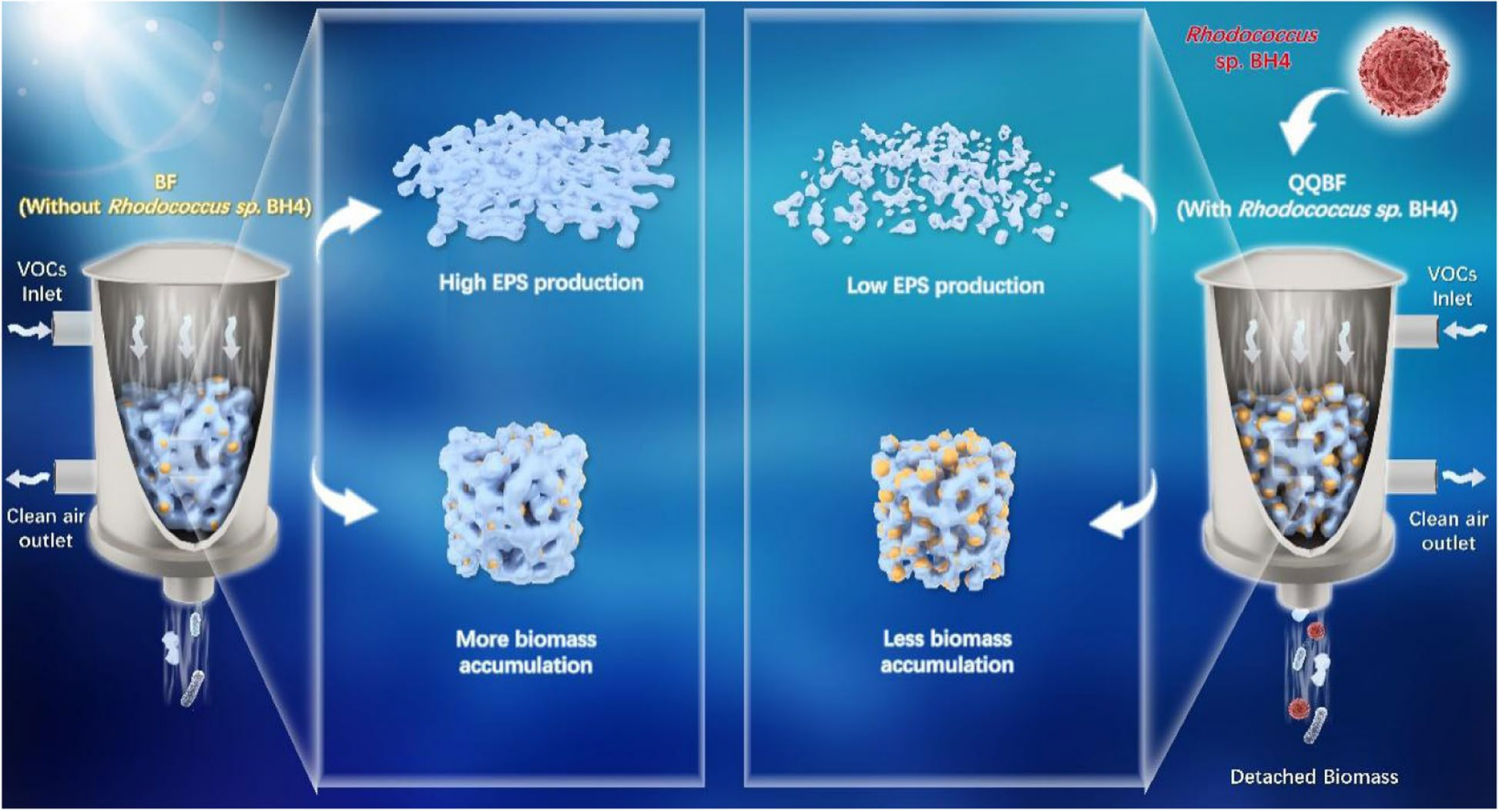

Video Abstract

**Supplementary Information:**

The online version contains supplementary material available at 10.1186/s40168-022-01345-5.

## Introduction

In the past decades, biofiltration (BF) has been increasingly used for the control of volatile organic compounds (VOCs) because of its low operating cost and lack of secondary pollution (Table S1) [1–3]. Microorganisms immobilized on the filler surface convert VOCs to carbon and energy sources for microbial growth [4, 5]. However, the excessive biomass accumulation would result in filter clogging and an unstable performance during long-term operation, which is a major bottle neck to the application of biofilters.

Various technologies have been developed to control the excessive biomass accumulation in biofilters. Physical and chemical methods, including mechanical mixing and chemical reagent flushing, have demonstrated effectiveness in the removal of excess biomass from biofilters [6, 7]. However, a growing concern is that the activity of microorganisms is damaged and difficult to restore stable operation. Han et al. [8] found that the removal rate of gaseous toluene was reduced from 88 to 20% after using a mechanical mixing method to remove biomass. Cox and Deshusses [9] showed that the activity of microorganisms was accompanied by the complete loss while using H_2_O_2_ and NaClO to remove excessive biomass. These researches revealed different biomass control methods but more or less affect microbial activity. Furthermore, it has been established that decreased microbial activity temporarily inhibits operational performance [10]. Therefore, it is crucial to develop a novel strategy to control excessive biomass while maintaining a stable performance.

Recently, quorum quenching (QQ)-based biomass control strategies have attracted attention [11, 12]. QQ is the method through degradation of the signal molecules (*N*-acylhomoserine lactones (AHLs)) to inhibit bacterial communication and thus regulate certain group behaviors of microorganisms [13, 14]. Traditionally, there are two ways to inhibit QS, enzymatic QQ and bacterial QQ enzyme [13, 15, 16]. In our previous study, acylase (QQ enzyme) has been successfully applied to control excessive biomass in gas biofilters, inhibiting biofilm adhesion strength and reducing extracellular polymeric substances (EPS) secretion [11]. However, the easy inactivation of acylase could greatly limit its industrial application. Therefore, it is essential to find an alternative QQ method for controlling biomass in gas biofilters. *Rhodococcus* sp. BH4, a quorum quenching bacterium isolated from activated sludge, has shown great potential in the delay of biofilm formation in MBRs for wastewater treatment [17, 18]. However, to the best of our knowledge, it has not been reported whether *Rhodococcus* sp. BH4 can be applied to gas-phase biofiltration to control clogging. Furthermore, the influence of exogenous *Rhodococcus* sp. BH4 on the original microbial community interactions and functional genes remains to be explored. Therefore, it is critical to track the effect of *Rhodococcus* sp. BH4 on biomass accumulation, biofilm adhesion, and QS microorganism gene expression in gas biofilters.

The assembly and evolution of microbial communities during long-term operation may affect many behaviors in bioreactors, such as biofilm adhesion and biomass accumulation [19, 20]. Moreover, changes in the composition of activated sludge can cause uncertainties in biofilm formation and accumulation [21–24]. Therefore, it is necessary to monitor the effect of *Rhodococcus* sp*.* BH4 adding on the microbial community of gas biofilters. To date, the understanding of biofilm in terms of biodiversity and community structure in bioreactors was improved through various attempts [23, 25, 26]. The keystone species, co-occurrence patterns of taxa in biofilms, and their putative interactions in microbial communities can be both explored using ecological network analysis [27–30]. Therefore, it is important to track the biofilm community during long-term operation after the addition of *Rhodococcus* sp. BH4, which could help in better understanding influence mechanism of the QQ bacteria-based biomass control strategy. Furthermore, identifying changes in the keystone species in biofilms using ecological network analysis may provide valuable insights into bacterial assembly during the biomass control process.

This study aimed to reveal the influencing mechanism of *Rhodococcus* sp. BH4 on biofilm adhesion strength and prove the feasibility of using QQ bacteria in the biomass control of gas biofilters. The biofilm adhesion strength and formation rate under different ratios of *Rhodococcus* sp. BH4 adding were investigated. Removal performance, operational stability, biomass accumulation, and pressure drop were evaluated with and without the addition of *Rhodococcus* sp. BH4 in biofilters. Moreover, EPS secretion, biofilm adhesion strength on the filler surface, and quenching ability of AHLs at different periods were explored to confirm the effect of *Rhodococcus* sp. BH4 on biofilm characteristics. High-throughput sequencing and random matrix theory (RMT)-based phylogenetic molecular ecological networks (pMENs) were used to investigate the development of microbial communities and the differences in keystone species. Metagenomics was also employed to explore the influencing mechanism of *Rhodococcus* sp. BH4 on biofilm adhesion by comparing the differences in the expression of QS-related genes.

## Methods

### Biofilm formation and adhesion strength assay

The effect of *Rhodococcus* sp. BH4 on the reduction in activated sludge biofilm formation and adhesion strength was tested through biofilm formation assays. The *Rhodococcus* sp. BH4 used in this study was screened from activated sludge, which was provided by Lee et al. [13, 15]. Activated sludge from the Tianjin Jinnan Wastewater Treatment Plant (Tianjin, China) and incubated *Rhodococcus* sp*.* BH4 were both washed with buffered saline solution (0.9% NaCl, *pH* = 7), and the optical density (OD) values were set to 1.0 (the details are provided in the supporting information, Supplementary method S1). Subsequently, the *Rhodococcus* sp. BH4 was introduced into activated sludge at ratios of 0%, 10%, 20%, 30%, 40%, and 50%. Each mixed solution (100 mL) was incubated for 24 h in a 250 ml Erlenmeyer flask at 28 °C and 120 rpm. Each Erlenmeyer flask was placed with a wooden board (8 × 2 × 0.5 cm), and the wooden board was timed weighing to measure the biofilm accumulation and formation rate. The adhesion strength of the biofilm was analyzed as described in our previous study [11, 31]. Specifically, when microorganisms adhere to the surface, the biofilm interacts with the surface of the carrier, allowing biofilm adhesion. This process is related to the shear force of the water flow. When the shear force of water is sufficiently large, the biofilm is desorbed from the carrier surface. Biofilm adhesion strength was calculated using the magnitude of the shear force and the action time (supporting information, Supplementary method S1).

### Biofilter operations

Two laboratory-scale biofilters (BF and QQBF) were operated in parallel to treat toluene gas, as shown in Fig. S1. Both biofilters were made of acrylic glass, having a height of 45 cm and an inner diameter of 6 cm. Wooden balls (diameter 8 mm) were selected as fillers and were packed to a depth of 33 cm. The activated sludge collected from the Tianjin Jinnan Wastewater Treatment Plant was cultivated for 7 days using liquid toluene, as described by Wang et al. [32]. During the start-up period, BF was inoculated only with activated sludge, and QQBF was inoculated with a mixture of activated sludge and *Rhodococcus* sp*.* BH4 solution (30%). Air and liquid toluene were mixed to prepare gaseous toluene. Nutrient solution was regularly sprayed from the top into the biofilter; its formula is shown in Table S2. The inlet toluene concentration and gas flow of the two biofilters were both 400 ± 100 mg/m^3^ and 0.1 m^3^/h. More operating conditions are listed in Table S3. Removal efficiency, pressure drop, and biomass accumulation were continuously monitored to evaluate the performance of the biofilters.

### Determination of QQ-related activity in the biofilm

The QQ-related activity was estimated through measuring the degradation of exogenous AHL standards using bioassay [33]. The bioassay was based on the reported strain (*A. tumefaciens* A136), X-Gal (5-bromo-4-chloro-3-indolyl-*β*-d-galactopyranoside), and LB agar. C8-HSL (octanoyl l-homoserine lactone) was selected as the standard AHL because it has been detected in the biofilm in our previous study [31, 34]. The specific methods were as follows: first, the biofilm was collected from the two biofilters in the 25th, 45th, 65th, and 90th day. Then, the samples were washed with PBS (phosphate-buffered saline) buffer three times. A total of 1.0 g of each biofilm sample was weighed out and resuspended in an Erlenmeyer flask with 50 ml of PBS buffer. Meanwhile, C8-HSL was added into the flask, and the final concentration was 200 nM. The degradation of C8-HSL was analyzed to determine the QQ ability in the biofilm, which follows first-order reaction kinetics and could be fitted as the Eq. (1):

1 $${c}_t={c}_0\cdot {e}^{- kt}$$

where *c*_0_ and *c*_*t*_ are the C8-HSL concentration at 0 time and *t* time (nM), respectively; *k* is the QQ rate (min^−1^), and *t* is the sampling time (min).

### DNA extractions and 16S rDNA sequencing

The filler samples in the two biofilters were collected at each sampling point (the 25th, 45th, 65th, and 90th day). The biofilm on the filler surface was stripped under the ultrasonic vibration condition. Then the biofilm samples were centrifuged at 8000 rpm for 5 min, repeated three times. DNA from different samples was extracted using the E.Z.N.A. ®Stool DNA Kit (D4015, Omega, Inc., USA) according to manufacturer’s instructions. Amplicons were then used for high-throughput sequencing on NovaSeq PE250 platform at LC-Bio Technology Co., Ltd, Hang Zhou, Zhejiang Province, China. The description of data analysis process was shown in supporting information (Supplementary method S2). The raw reads obtained from the samples were deposited in the NCBI GenBank (accession number: PRJNA818815).

Alpha diversity indices including Shannon’s diversity index and Pielou’s index were calculated using the program QIIME [35]. Kruskal-Wallis test was used to find the biomarkers that differ significantly at the phylum level in microbial communities (*p* < 0.01) [29]. To compare the species composition between samples, nonmetric multidimensional scaling (NMDS) based on Bray−Curtis dissimilarity distance matrices was calculated using OTUs table [36].

### Network construction using the RMT-based approach

The pMENs of the microbial communities in BF and QQBF samples during the operation were constructed on a comprehensive molecular ecological network analysis pipeline (http://ieg2.ou.edu/MENA) through random matrix theory (RMT)-based methods [28]. A correlation matrix was obtained using OTUs relative abundance, which was then converted to adjacency matrix based on Pearson correlation analysis. Fast greedy modularity optimization was used to separate the networks into multiple dense modules [27]. Besides, the Maslov-Sneppen method was used to construct corresponding random networks of each pMEN for comparison [37]. Within-module connectivity (Zi) and among-module connectivity (Pi) were calculated to identify the keystone species [38]. Also, the small-world property of the networks was analyzed through calculating the small-world coefficient (*σ*) [39]. Gephi (WebAtlas, France) was used for network visualization [29].

### Metagenomics sequencing analysis

Microbial samples were taken from BF and QQBF on the 90th day for metagenomic sequencing. The relative abundances of QS pathway, protein, and polysaccharide synthesis pathway genes of QQBF and BF samples were compared using the Kyoto Encyclopedia of Genes and Genomes (KEGG) database annotation to explore the possible effect mechanism. A local QS database was set up to search for QS-related bacteria based on UniProtKB, according to Xu et al [23]. The raw reads obtained from the samples were deposited in the NCBI Sequence Read Archive (SRA) database (accession number: PRJNA818845). More details about DNA extraction, DNA library construction, and data analysis are provided in supporting information (Supplementary method S3).

### Analytical methods

The concentration of EPS in the biofilm was analyzed on the 25th, 45th, 65th, and 90th day. Protein (PN) and polysaccharide (PS) contents were determined using the Lowry method and phenol sulfuric acid method, respectively [34]. The concentration of gaseous toluene was determined using a gas chromatograph (GC7900, Tianmei, China), according to a previous study [40]. Biomass accumulation in biofilters was measured using a modified weighing method [41]. A laser scanning confocal microscopy (CLSM; Leica Microsystems, Germany) was used for biofilm visualization on the filler surface [11]. The biofilm adhesion strength on the filler surface between the two biofilters was compared using ultrasonic detachment efficiency. Mixed liquor suspended solids (MLSS) were measured using standard methods [42]. The pressure drop of the filter bed was measured regularly using a piezometer (JJG540-88, Bolaite, Shanghai, China). The calculation of the biofilter performance and pressure drop curve-fitting methods is given in supporting information (Supplementary method S4).

Statistical differences were analyzed by one-way analysis of variance (ANOVA) using SPSS (version 19.0; SPSS Inc., Chicago, Illinois, USA), and *p* < 0.05 was considered to be the threshold of statistical significance.

## Results

### Effect of Rhodococcus sp. BH4 on biofilm adhesion strength

**Fig. 1:**
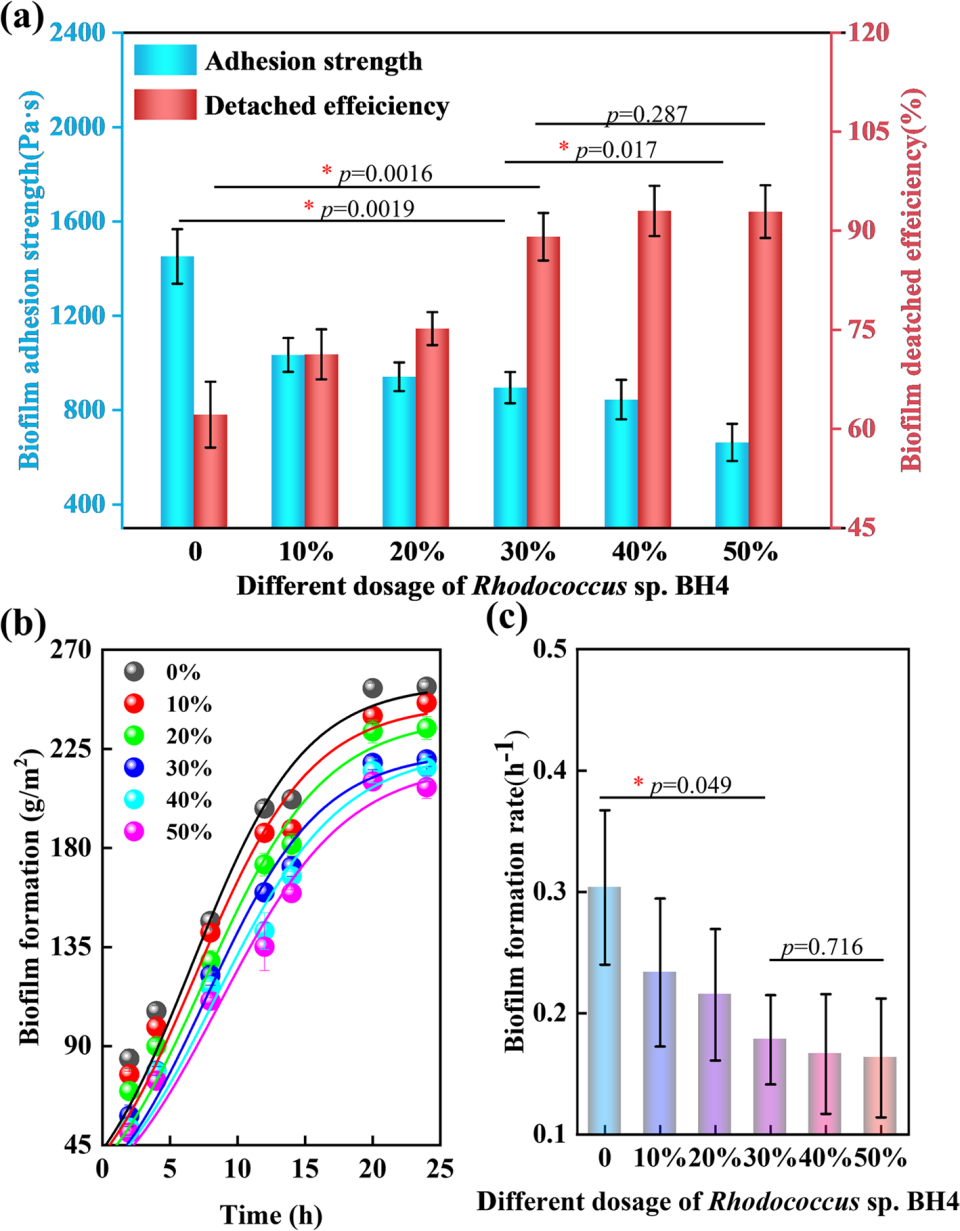
Effect of different dosage of *Rhodococcus* sp. BH4 on biofilm characteristics. **a** Biofilm adhesion strength and detachment efficiency. Detachment efficiency is calculated using the ratio of detached biofilm to total biofilm formation. Fitting results of **b** biofilm formation and **c** biofilm formation rates using logistic model. Error bar, standard deviation (*n* = 3)

The effects of *Rhodococcus* sp*.* BH4 on biofilm adhesion strength is shown in Fig. 1a. A clear dose response was observed for *Rhodococcus* sp. BH4 ratios ranging from 10 to 50%. It was found that the biofilm adhesion strength decreased gradually with an increase in *Rhodococcus* sp*.* BH4 dose. As compared to the control (in which only activated sludge was present), the biofilm adhesion strength decreased by approximately 38% and 54% for *Rhodococcus* sp*.* BH4 ratios of 30% and 50%, respectively. Meanwhile, the biofilm detachment efficiency (at the end of the biofilm adhesion test) gradually increased. Besides, the activated sludge biofilm formation was tested using different ratios of *Rhodococcus* sp. BH4 over a 24-h period, as shown in Fig. 1b and c. This was similar to the adhesion test, in which biofilm formation decreased gradually with an increase in *Rhodococcus* sp*.* BH4 dose (Fig. S3). The biofilm formation curve was fitted using a logistic model to determine the rate of biofilm formation. It was found that the biofilm formation rate was 0.18 h^−1^ and 0.16 h^−1^ for *Rhodococcus* sp*.* BH4 ratios of 30% and 50%, respectively, compared to the control (0.30 h^−1^). These results suggested that biofilm adhesion and formation on the carrier surface were closely related to the proportion of *Rhodococcus* sp. BH4. Therefore, to avoid the influence of excess *Rhodococcus* sp. BH4 on the performance of the biofilter, activated sludge containing 30% *Rhodococcus* sp. BH4 was used for biofilter start-up.

### Effect of Rhodococcus sp. BH4 on biofilter performance

**Fig. 2:**
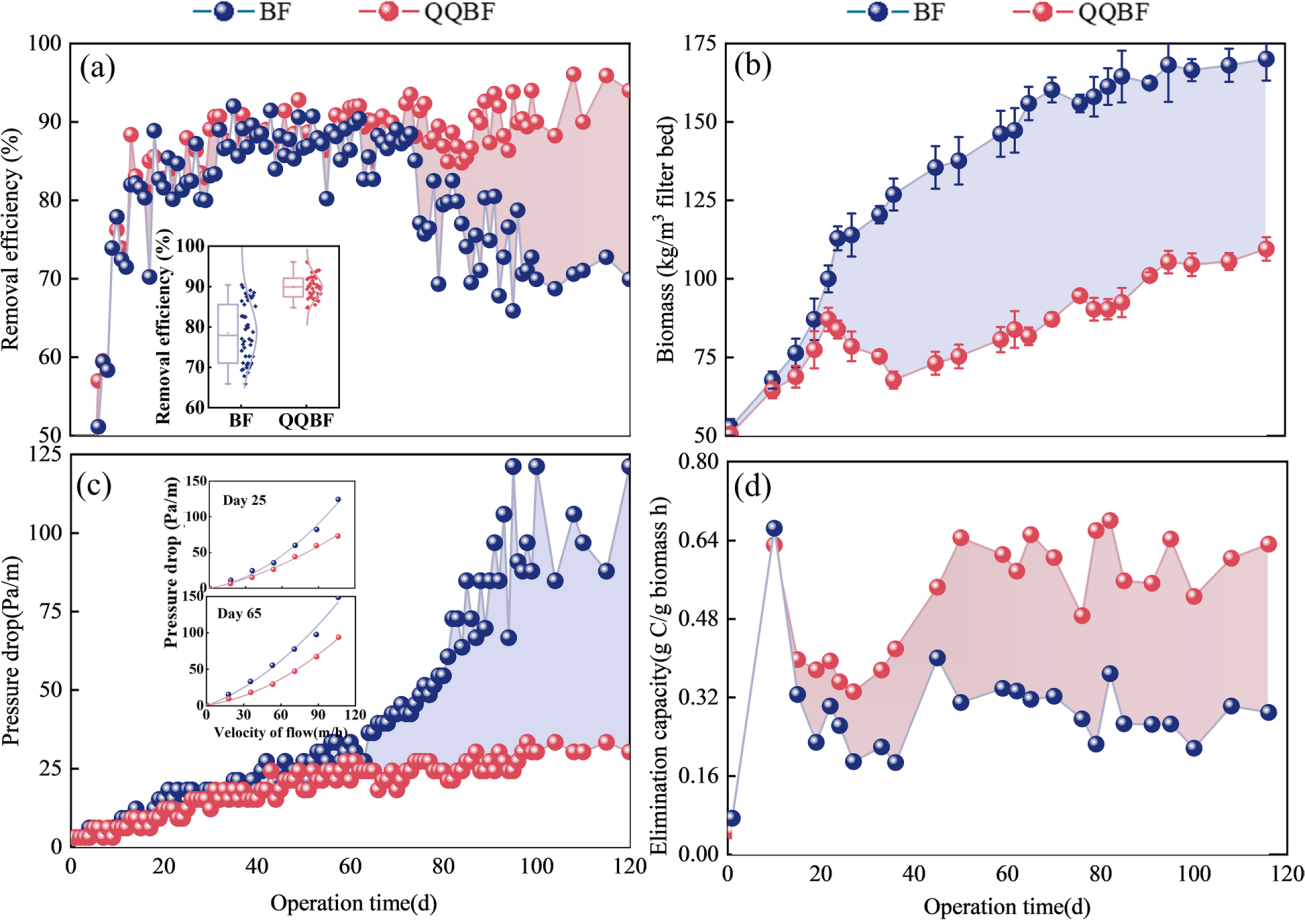
Reactor performance of BF and QQBF during the 120 days of operation. **a** Removal efficiency of gaseous toluene (inset represents the operation stability analysis from day 60 to day 120). **b** Biomass accumulation (mean ± SD (*n* = 3)). **c** Pressure drop (inset represents the Ergun equation fitted curves of pressure drop for BF and QQBF on the 25th and 65th day). **d** Elimination capacity

To evaluate the effect of *Rhodococcus* sp. BH4 on biomass control and operational stability, two biofilters treating gaseous toluene were operated for 120 days, of which QQBF was supplemented with *Rhodococcus* sp. BH4 (30%). As shown in Fig. 2a, the removal efficiency (RE) of BF and QQBF presented similar trends for the first 60 days, which all remained above 80%. However, the RE of BF declined gradually, while the RE of QQBF was stable from day 70 to day 120. The operational stability analysis during this period demonstrated that the RE of BF ranged from 63 to 92%, whereas the RE of QQBF ranged from 85 to 96%. It is suggested that the operational stability was greatly improved with the addition of *Rhodococcus* sp. BH4 in QQBF compared with that in the BF. Meanwhile, biomass accumulation in BF and QQBF showed significant differences during the operation (Fig. 2b). From day 20, the biomass accumulation in QQBF fluctuated and increased gradually (109 kg/m^3^ filter bed), whereas BF increased rapidly and reached 170 kg/m^3^ filter bed on the 116th day. This indicated that the addition of *Rhodococcus* sp. BH4 in QQBF reduced biomass accumulation by 36%, as compared to BF.

The monitoring results for the filter-bed pressure drop are shown in Fig. 2c. The pressure drop in BF increased rapidly and fluctuated after day 60 to reach 121 Pa/m, whereas QQBF remained a low pressure drop and only 30 Pa/m on the 120th day. In addition, the pressure drop and flow rate curves on the 25th day and 65th day were fitted using the Ergun equation (Fig. 2c, inset). Table S4 shows the fitting equation between the flow rate and pressure drop. Regression parameters (*α* and *β*) were used to indicate the clogging status. It can be found that the difference in the fitted curve appeared on the 25th day. The value of the parameters (*α*) in BF and QQBF were 0.36 and 0.26, respectively. Furthermore, *α* increased to 0.63 and 0.29 in BF and QQBF, respectively, on day 65. This indicated that the addition of *Rhodococcus* sp. BH4 improved the structure of the filter bed in the early stage and maintained a low pressure drop in QQBF during operation. It is reported that during the long-term operation, the removal efficiency would be decreased duo to the excessive biomass accumulation and increased pressure drop [8, 11], which explained the significantly decreased removal efficiency in BF after 60 days. Therefore, the addition of *Rhodococcus* sp. BH4 controlled the clogging in QQBF and maintained stable operation. Moreover, lower biomass accumulation in QQBF increased the elimination capacity per unit biomass (see Eq. S7 for calculation) by nearly twofold (Fig. 2d). These phenomena indicated that the addition of *Rhodococcus* sp. BH4 significantly improved the overall performance of the QQBF.

### Effect of Rhodococcus sp. BH4 on biofilm characteristics in the biofilter

**Fig. 3:**
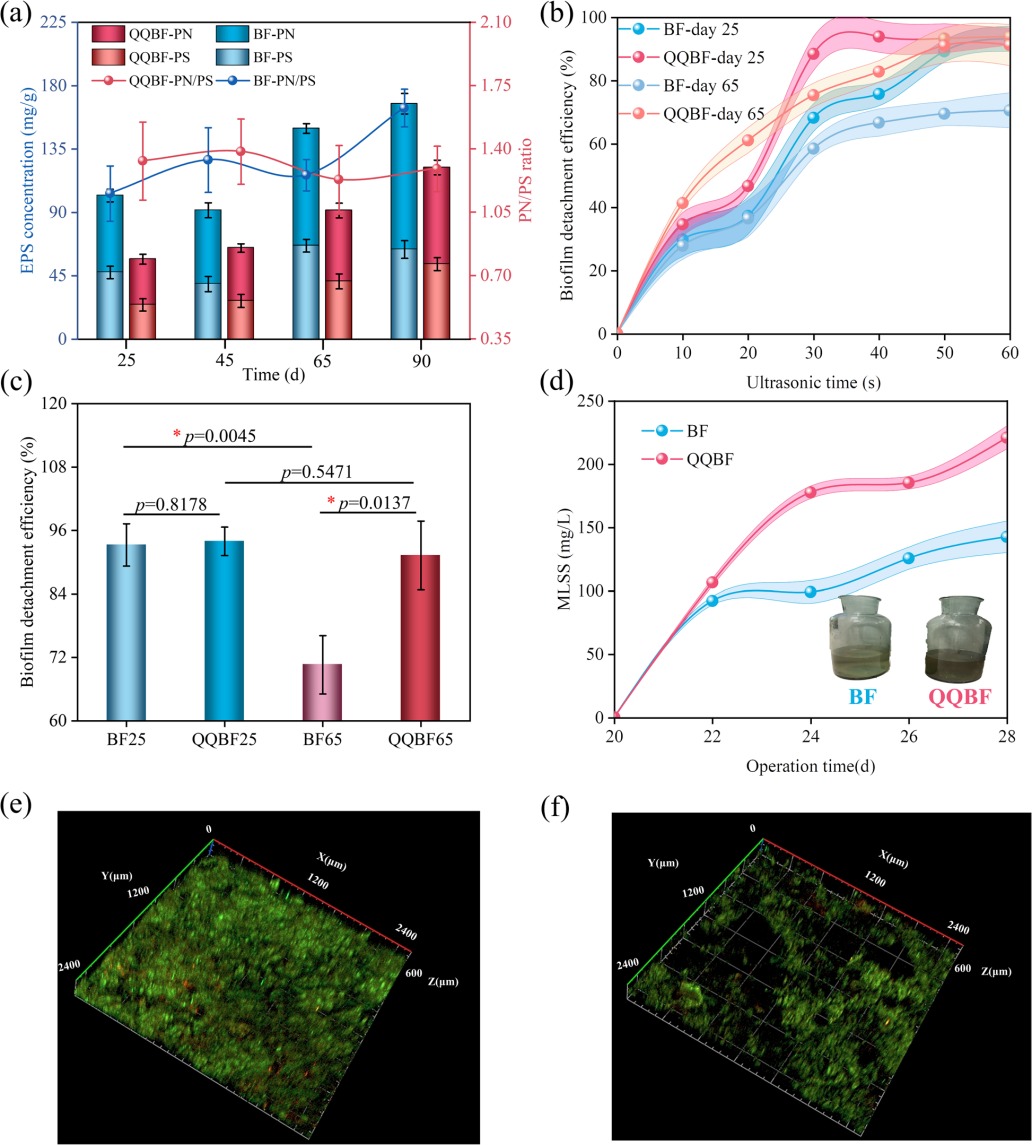
**a** EPS contents in BF and QQBF biofilms during the whole operation. **b** Biofilm adhesion strength comparison on the filler surface in BF and QQBF on the 25th day and 65th day (the adhesion was compared by the detachment efficiency of biofilms during ultrasonic cleaning). **c** Biofilm detachment efficiency at the end of ultrasonic cleaning (60 s). The star symbol represents significant difference (*p* < 0.05). **d** MLSS concentration in leachate from BF and QQBF for one cycle (day 20 to day 28). Insets represent actual leachate conditions for BF and QQBF at the end of the cycle. CLSM images of biofilm formed on the filler surface in **e** BF and **f** QQBF after the 65-day operation. Green and red indicate live microbial cells and dead microbial cells, respectively. Error bar, standard deviation (*n* = 3)

The EPS content of the biofilm in biofilters was determined during the operation. The concentrations of PN and PS were all increased with operation in BF and QQBF (Fig. 3a). However, at each stage, both PN and PS were lower in QQBF than in BF (*p* < 0.05; Table S5). Previous studies have shown that PN and PS play important roles in biofilm adhesion [11, 31]. Therefore, decreased EPS secretion in QQBF could have reduced the biofilm adhesion strength to the filler surface.

The fillers in BF and QQBF were taken out on the 25th day and 65th day, and the attached biofilms to fillers were detached using an ultrasonic cleaning machine to compare the biofilm adhesion stability on the filler surface. After 60 s of ultrasonic treatment, the biofilm detachment efficiency on the 25th day in both BF and QQBF was more than 90% (Fig. 3c) and showed no significant difference (*p* = 0.82). However, the stable 90% biofilm detachment efficiency of QQBF at 30 s (70% in BF) continued to show low adhesion strength (Fig. 3b). Moreover, the biofilm detachment efficiency on the 65th day in BF (70%) and QQBF (95%) was significantly different (*p* < 0.05). This suggested that biofilm adhesion in QQBF was inhibited by the addition of *Rhodococcus* sp. BH4, as compared to BF.

The MLSS concentrations accumulated in the leachate obtained from BF and QQBF were tested and compared (Fig. 3d). The MLSS concentrations increased with the operation in both BF and QQBF, owing to the regular spraying of the nutrient solution and the continuously detached biofilm. Interestingly, the MLSS content (220 mg/L) in QQBF was higher than that (140 mg/L) in BF (*p* < 0.05), and it was nearly two-fold on the 28th day. This phenomenon was also observed in the quenching activities (quenching rates of AHLs) of BF and QQBF (Fig. S4a). All the AHL quenching rates of QQBF biofilm samples were higher than those of BF for different periods (Fig. S4 and Table S6). Previous studies reported that the balance between native QQ bacteria and QS bacteria maintained a stable ecosystem [34], which explained the quenching activity of BF biofilms. Moreover, these results suggested that AHLs in biofilms were strongly degraded by *Rhodococcus* sp. BH4 (Fig. S4b), which affected the balance of the native ecosystem and inhibited biofilm adhesion. Meanwhile, the sparse biofilm distribution on the QQBF filler surface, as visualized by CLSM, also illustrated the lower biomass, as compared to BF (Fig. 3e and f). A dense accumulation of biofilm was observed in BF, which almost covered the filler surface. However, the biofilm accumulation in QQBF was thinner and sparser, indicating that less biomass was attached to the filler surface. This may be due to the unstable adhesion of biofilm in QQBF, leading to the detachment of biomass from filler surface into the leachate.

**Fig. 4:**
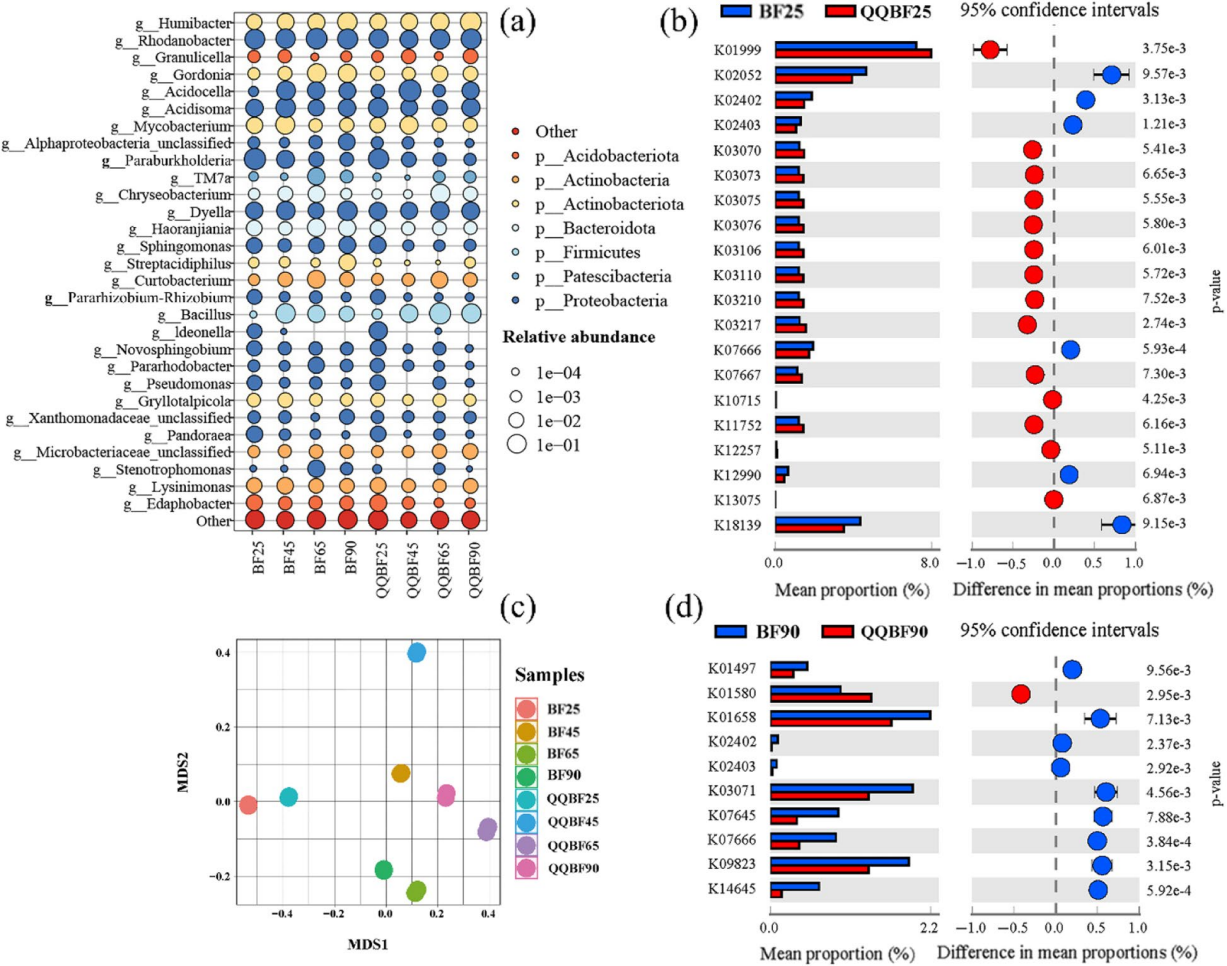
**a** Relative abundance of the top 30 genera in BF and QQBF biofilm samples during the whole operation. Significantly different (*p* < 0.01) QS-related genes in BF and QQBF biofilm samples on **b** day 25 and **d** day 90. **c** Dissimilarities among the biofilm samples in BF and QQBF presented by NMDS analysis based on the Bray−Curtis distance

Functional gene prediction analysis was used to compare the relative abundances of QS genes. The QS-related genes in BF and QQBF samples were screened through KEGG metabolic pathway (Table S7), after which the significantly different genes were analyzed based on ANOVA one-way test (*p* < 0.01) (Fig. 4b and d). During the initial stage of biofilter operation (day 25), functional genes (detailed description in Table S7), such as K02052, K02402, and K18139 were higher in BF than in QQBF (*p* < 0.01). In contrast, some genes (K01999, K03070, K03073, etc.) showed a relatively lower relative abundance in BF. This suggested that the relative abundance of QS genes did not show a clear trend in BF and QQBF (Fig. 4b). Interestingly, this phenomenon disappeared on day 90; most of the significantly different QS genes presented a higher trend (*p* < 0.01) in BF, except K01580. This demonstrated that during the long-term evolution of biofilms, QS genes in QQBF biofilms were inhibited. It was confirmed that the biofilm formation and adhesion are closely related to the QS system in microorganisms [13, 34]. Hence, these results strongly indicate that the inhibited QS activity in QQBF affects the biofilm adhesion strength and makes it easy to detach, thereby reducing biomass accumulation and controlling clogging.

### Evolution of microbial community succession in BF and QQBF

A total of 1, 131, and 510 high-quality reads from the BF and QQBF biofilm samples were generated using 16S rDNA high-throughput sequencing. It was found that the microbial community in BF and QQBF showed significant between-group differences based on the analysis of similarities (ANOSIM; *R* = 1, *p* = 0.001; Fig. S5) and alpha diversity analysis (Fig. S6). This revealed that the addition of *Rhodococcus* sp. BH4 affected the assembly of microbial communities. Generally, Proteobacteria were the most abundant phylum (> 50%) in most biofilm samples (Fig. S7). The genera related to aromatic compound degradation, such as *Dyella*, *Pandoraea*, *Sphingomonas*, and *Rhodanobacter*, presented high relative abundance (21–42%, Fig. 4a) [43–45], which might maintain the degradation of toluene. Besides, Patescibacteria, Firmicutes, Actinobacteria, Actinobacteriota, and Acidobacteriota constituted the other dominant phyla in the biofilms (Fig. 4a).

Among these phyla, the evolution of Firmicutes showed different trends during operation in BF and QQBF (Fig. S7). The relative abundance of Firmicutes in BF and QQBF on day 45 was 15% and 5%, respectively; on day 65, it was 5% and 40% in BF and QQBF, respectively. This suggested that the microbial community was remarkably different between days 45 and 65. The NMDS analysis also showed similar results (Fig. 4c). It should be noted that microbial communities have a self-regulating ability to maintain system stability [46–48], and Firmicutes were previously reported to maintain biofilm stability [49, 50]. Therefore, a higher relative abundance of Firmicutes (especially *Chryseobacterium* genus) in BF at an early stage (days 25 and 45) may be used to enhance the construction of biofilm systems. On day 65, the increased relative abundance of Firmicutes in QQBF may be used to resist the effect (destabilize the adhesion) of *Rhodococcus* sp. BH4 on biofilm. Moreover, the QS-related genera in the BF and QQBF biofilms were 39% and 44%, respectively, on day 25. On day 90, they were 65% and 53%, respectively (Fig. S8). These results were consistent with QS-related gene abundance on days 25 and 90 (Fig. 4b and d), indicating that the addition of *Rhodococcus* sp. BH4 may inhibit the activity of QS microorganisms.

### Network topology features of bacterial communities in BF and QQBF

**Fig. 5:**
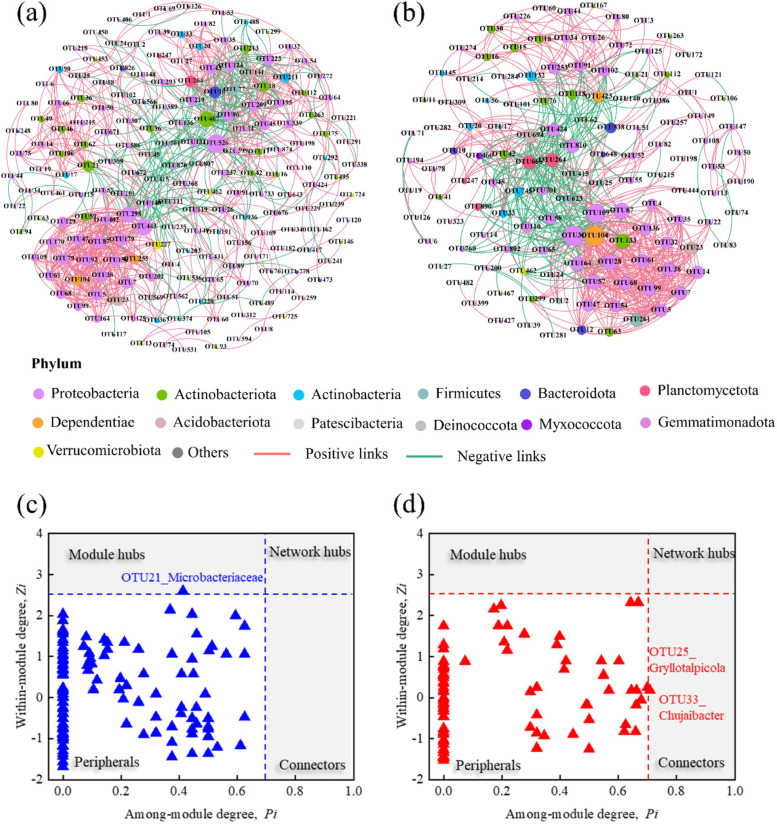
Phylogenetic molecular ecological networks (pMENs) of biofilm communities in **a** BF and **b** QQBF during the whole operation. The sizes of nodes and OUT labels are proportional to their node degrees and average relative abundance, respectively. Gray and red edges represent positive and negative interactions, respectively. The edge thickness is proportional to the absolute value of the correlation coefficient. Z-P plots represent the topological roles of species in **c** BF and **d** QQBF

Two RMT-based networks were established to analyze the ecological interactions of species in BF and QQBF biofilms (Fig. 5a and b). The major topological parameters of these networks, such as average path distance, clustering coefficients, and modularity, were significantly higher than those of the corresponding random networks; all the small-world coefficients (σ) were greater than one (Table S8). These results strongly indicated that the networks were nonrandom, and that pMENs possessed small-world and modular structures, regardless of BF and QQBF biofilms [27, 51]. Moreover, lower network nodes (130) and edges (705) indicated a simple topology in QQBF biofilms compared with BF biofilms (nodes: 189; edges: 912) (Table S8) [52]. Intriguingly, both BF (75%) and QQBF (70.5%) biofilms showed great species-species associations, but QQBF biofilms had a higher negative interaction (29.5%). The high negative interaction in QQBF might be related to the reduced abundance of QS species (Fig. S8) because QS plays an important role in species communication [53].

Module hubs and connectors are regarded as keystone taxa that drive biofilm succession in the biofilm ecological process. Therefore, within-module connectivity (*Zi*) and connectivity among modules (*Pi*) were used to identify the topological roles in the BF and QQBF biofilms (Fig. 5c and d). Most of the OTUs in the networks were peripherals, and there were no network hubs in the BF and QQBF samples (Fig. 5c and d). Only one module hub (OTU21) was considered keystone taxa that drive the biofilm succession in BF (Table S9). In QQBF, two connectors (OTU25 and 33) were identified instead of module hubs, which indicated a difference in the succession of BF and QQBF biofilms. OTU21 (*f_Microbacteriaceae*), which has been isolated from many natural environments, is indicating its importance in environmental ecological cycles [54, 55]. Moreover, the function of aromatic compounds degradation [56] and biological growth promotion [57] demonstrates its important role in BF biofilms. Intriguingly, OTU25 (*g_Gryllotalpicola*) and OTU33 (*g_Chujaibacter*) in QQBF both responded to exogenous stress and the function of aromatic compounds degradation [58]. These results suggest that the keystone taxa were not only affected by the degradation of pollutants but were also affected by external factors, such as the addition of *Rhodococcus* sp. BH4 in QQBF. Furthermore, the keystone taxa in the BF and QQBF accounted for extremely low abundance (Table S10, average 0.22–0.34%), indicating that the key roles (such as maintaining a stable community and biofilm formation) played by low-abundance species should not be ignored.

### Mechanism of Rhodococcus sp. BH4 on biofilm adhesion strength in QQBF

**Fig. 6:**
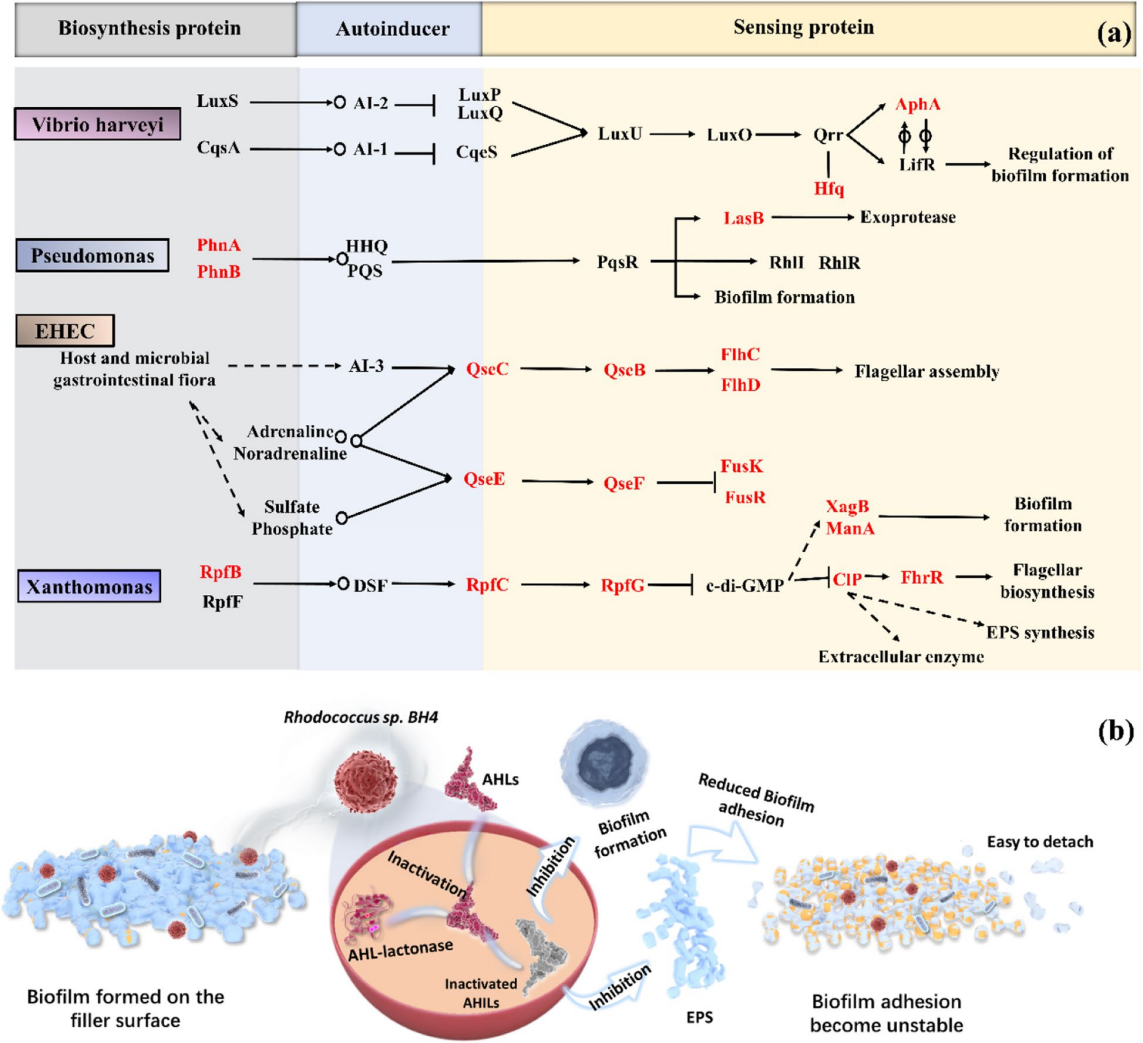
**a** QS regulatory pathways of typical QS microorganisms in BF and QQBF biofilm samples based on metagenomic analysis (red represents upregulated relative abundance in BF compared to QQBF). The details about the gene description and relative abundance are presented in the supporting information, Table S11). **b** Possible molecular mechanism of *Rhodococcus* sp. BH4 regulating biofilm adhesion strength in biofilters

Gene expressions in BF and QQBF biofilms on day 90 were analyzed using metagenomic sequencing. The total gene expression levels (Fig. S9) and the number of upregulated and downregulated genes (Fig. S10) did not show significant differences between BF and QQBF biofilms. Meanwhile, QS-related behaviors, such as biofilm formation and bacterial secretion system, were annotated as significantly enriched differential genes in the KEGG database (Fig. S11). Therefore, the differential genes in the microbial QS system pathways were annotated using the KEGG database (Fig. 6a; Table S11). The results showed that differential QS genes in typical QS microorganisms were downregulated in QQBF biofilms, whereas no QS genes were upregulated (Fig. S12). In addition, the final functions of these QS pathways were related to biofilm formation or EPS synthesis, suggesting that this might be a possible path to reduce biofilm adhesion. Moreover, the majority of QS genes that were downregulated were annotated as sensing proteins, indicating that their effects could occur during the sensing process of signaling molecules. A possible mechanism of action of *Rhodococcus* sp. BH4 on biofilm adhesion was proposed based on these results (Fig. 6b)*.* The AHLs in biofilms were degraded by the AHL lactonase, an endoenzyme produced from *Rhodococcus* sp*.* BH4 [59], and then, the QS activity was inhibited, and QS genes were downregulated, thereby reducing biofilm formation and EPS secretion, finally leading to a decrease in the biofilm adhesion strength.

## Discussion

This study improved the operational performance of gas biofilters by reducing the adhesion of biofilms. Moreover, the effect of QQ bacteria (*Rhodococcus* sp*.* BH4) on biofilm characteristics was analyzed from the perspective of community assembly and evolution and combined with the analysis of functional gene differences. The results of this study provided insights into the possible mechanisms of reduced biofilm adhesion strength.

### Microbial influence on biofilm formation and accumulation

In this study, activated sludge biofilm formation was reduced by using different ratios of *Rhodococcus* sp. BH4 to inhibit cell adhesion (Fig. 1). More analysis found that the results were completed through the inhibition of QS-related behavior and decreased EPS secretion, which was confirmed in previous studies [12, 31]. It was reported that the biofilm formation and inhibition could be affected by AHLs concentration and EPS production [60, 61]. Therefore, the decrease in the relative abundances of QS-related species and genes (Fig. 4 and S8) in QQBF biofilm (compared to the BF biofilm) corresponded to its low biomass (Fig. 2), which also validated the association between QS behavior and biofilm accumulation.

Meanwhile, positive interactions dominated both biofilm systems (Fig. 4), indicating the importance of cooperative behaviors in biofilm formation. Coaggregation and co-colonization were regarded as cooperative behaviors that improved biofilm formation [62]. Furthermore, increased microbial resistance and cooperative reciprocity among bacteria were closely linked [62]. Therefore, the stability of biofilm in QQBF might be decreased due to the highly negative interactions between species (Fig. 5). Additionally, keystone species played a non-negligible role in biofilm formation [23]. During the biofilm formation stage, some keystone species acted as pioneers and facilitated the formation and adhesion of biofilms on the carrier surface. These pioneers disappeared during the biofilm accumulation stage, indicating selective succession of biofilms [19, 63]. In this study, the differences in keystone species in BF and QQBF biofilms (Fig. 5) showed that the accumulation and evolution of biofilms were limited by environmental factors. The keystone species in BF was identified as *f_Microbacteriaceae*, which was found a great potential function in aromatic compound degradation and biological growth promotion [56, 57]. Meanwhile, the keystone species in QQBF were identified as *g_Gryllotalpicola* (f_Firmicutes) and *g_Chujaibacter*, which exhibited the ability to respond to environmental stress, such as maintenance of biofilm stability [49, 50, 58]. These results suggested the possible relationship between microbial community evolution with environmental factors and provide an additional explanation for the presence of higher biomass in BF than that in QQBF.

### Relationship between biomass and performance of gas biofilters

The pollutant removal performance of biofilm-based biofilters was closely related to their biomass accumulation [7, 64, 65]. During the start-up phase, biofilms were expected to achieve high growth rates to meet the pollutant removal requirements. In this study, biomass accumulation in BF and QQBF improved two-folds approximately in the first 20 days (Fig. 2b) and reached a high gaseous toluene removal efficiency (Fig. 2a). However, in BF, the operation performance decreased and became unstable after 60 days with continuously growing biomass and high-pressure drop. This suggested that the unlimited growth of biofilms in bioreactors did not improve the operational performance but led to filter clogging. This phenomenon was also manifested in sequencing batch reactor (SBR) and membrane bioreactor (MBR) [29, 66, 67], indicating that excessive biomass/biofilm growth was a common problem in bioreactors. Interestingly, this study revealed that low biomass accumulation in QQBF (Fig. 2) improved the operation stability and decreased the pressure drop, which demonstrated the necessity and effectiveness of controlling excess biomass accumulation. Additionally, regularly discharged excess sludge in the SBR prevented sludge bulking and maintained stable operation [68]. Regular cleaning of the membrane surface can reduce membrane pressure to maintain low-energy consumption and operational stability [69]. Notably, all these methods of improving operational stability were reflected in the middle and later stages of operation, suggesting a difference in the relationship between biomass and performance during the early and late operation stages. Moreover, since the *Rhodococcus* sp. BH4 have no ability to degrade toluene (Fig. S13), the high operation stability in QQBF was caused by the less biomass accumulation and decreased pressure drop. Therefore, all these results showed that keeping the biomass balance could help improve the operational stability in bioreactors.

### Significance of application of Rhodococcus sp. BH4 in gas biofilters

**Fig. 7:**
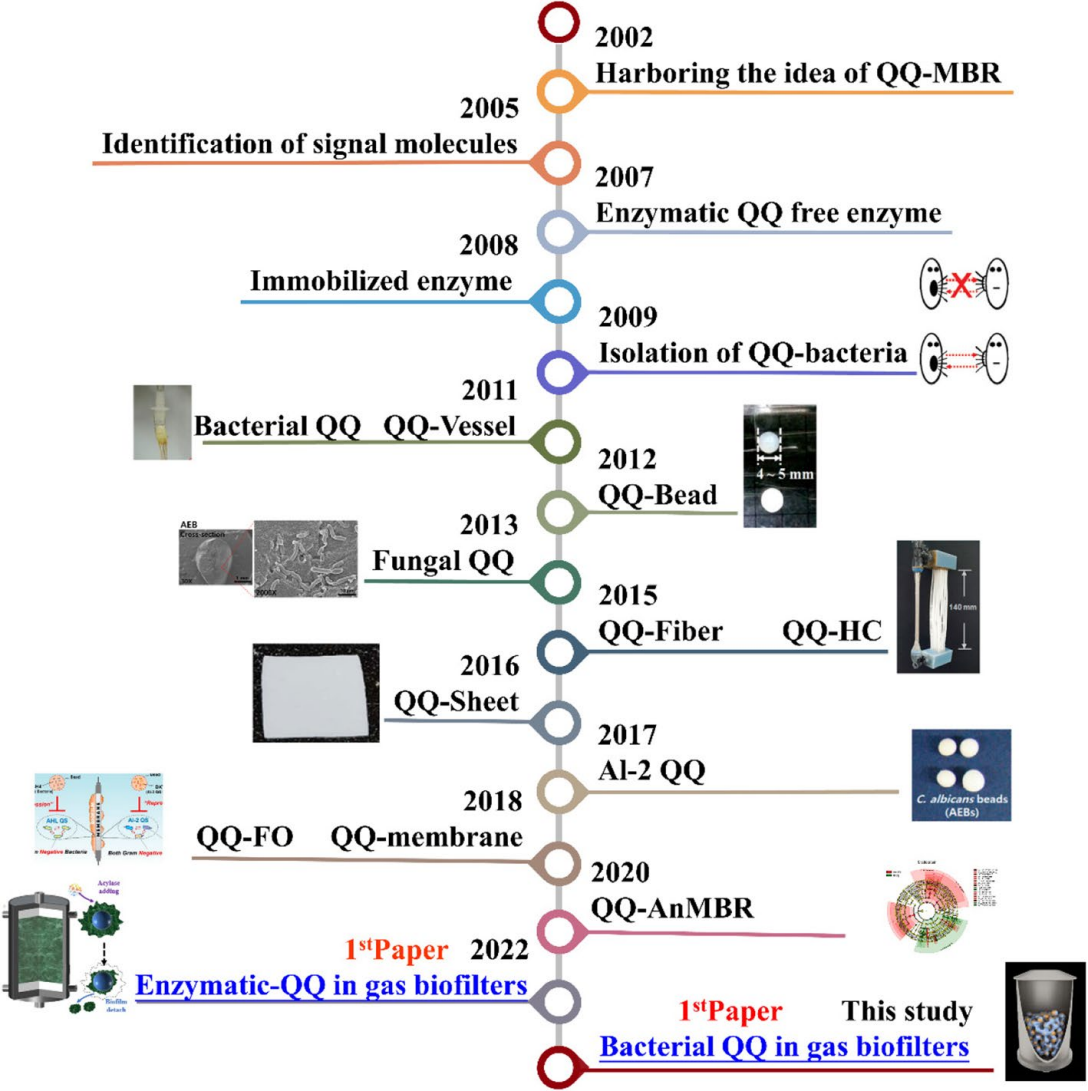
Development of QQ technology application in environmental bioreactors

Since the first application of biotechnology in the treatment of waste gas in the 1950s, many researches have been devoted to its difficult problems in engineering applications (Table S1). Among this, excessive growth of biofilms has always been a limiting factor for stable operation in bioreactors [6, 13, 69]. Various methods have been developed to solve this problem but not satisfactory [5, 9]. Since its discovery in 2009, the problem of MBR biofouling has been solved by inhibiting QS behavior. This has led to the rapid development of QQ-based biofilm growth control technology (Fig. 7) [15, 70, 71]. However, almost all biofouling control technologies that use QQ enzymes and QQ bacteria are concentrated in MBR (Table S12) [15, 72, 73]. This study aimed to assess if QQ technology could solve the problem of biofilm growth in other types of bioreactors. Excessive biomass accumulation in gas biofilters is a major operational obstacle; it leads to clogging, reduces the overall performance, and increases energy consumption [5–7]. This problem seemed possible to be controlled by inhibiting the QS system in biofilms [11]. In contrast to the water-phase biofilm, the biofilm in the gas biofilter remains in the gas-solid phase system for longer periods [32]. The microbial community evolution, functional genes, and keystone species in gas biofilters might vary from MBR. Therefore, the effect of QQ technology on gas biofilters is unknown, especially with the addition of QQ bacteria. This study focused on the effects of QQ bacteria (*Rhodococcus* sp. BH4) on gas biofilter clogging and found that it decreased biofilm adhesion strength to reduce biomass accumulation. Moreover, QS gene expression in biofilms was inhibited, and the keystone species were found to vary. These results showed the effectiveness of controlling excess biomass accumulation through adding QQ bacteria.

## Conclusion

In this study, the filter-bed clogging of waste gas biofilters during the long-term operation was prevented by using the QQ bacteria (*Rhodococcus* sp. BH4). After a 120-day operation, the biomass accumulation in QQBF (adding *Rhodococcus* sp. BH4) was found reduced by 36%, compared to BF. Meanwhile, the filter-bed pressure drop was found only 30 Pa/m in QQBF but 121 Pa/m in BF at the end of the operation, which was also confirmed through the Ergun equation. Additionally, the operational stability was significantly improved in QQBF due to the control of clogging. Compared to BF, the QQBF biofilm had lower adhesion strength and decreased EPS production, leading to easier detachment of biomass from filler surface into the leachate. Moreover, it was revealed that the relative abundance of QS-related species and function genes in QQBF was lower than that of BF through 16S rDNA gene sequencing and metagenomic sequencing analysis. The keystone species in QQBF was found a function to keep biofilm community stability through RMT-based network analysis. Finally, the results of KEGG database annotation based on metagenomic sequencing analysis indicated that the functional genes in QS pathway were inhibited, and thus, EPS secretion and biofilm formation were decreased, which reduced the biofilm adhesion. Overall, this is the first study that achieved biomass control while maintaining stable performance (without interrupting operation) through using QQ bacteria in gas biofilters, which provided new insights into clogging control and the application of QQ technology in bioreactors.

## Supplementary Information

**Additional file 1: Figure S1.** The schematic diagram of biofilters setup. **Figure S2.** Schematic diagram of biofilm adhesion strength test. **Figure S3.** Effect of different dosage of *Rhodococcus sp*.BH4 on biofilm formation. **Figure S4.** (a) AHL quenching rate and (b) quorum quenching activity towards the exogenous AHL in the biofilm samples on Days 25, 45, 65 and 90. **Figure S5.** The sample distance matrix obtained based on the bray Curtis algorithm. The results were used to test whether the difference between groups is significantly greater than the difference within the group. **Figure S6.** Chao1 index, number of OTUs, Shannon index and pielou_e index in BF and QQBF biofilms. **Figure S7.** Relative abundance of biomarkers at the phylum level in (a) BF and (b) QQBF. The biomarkers were chosen through Kruskal-Wallis test. **Figure S8.** Comparison of relative abundance of QS-related bacteria in BF and QQBF biofilms at day 25 and 90. **Figure S9.** Comparison of gene expression levels in microbial samples at day 90 between BF and QQBF based on metagenomic analysis. **Figure S10.** Comparison of the number of up-regulated and down-regulated genes in microbial samples (BF vs QQBF) at day 90 of BF and QQBF based on metagenomic analysis. **Figure S11.** Differential gene enrichment results in microbial samples at day 90 of BF and QQBF based on KEGG database annotations. **Figure S12.** Up-regulation of QS genes in BF biofilm samples compared to QQBF in QS regulatory pathways based on metagenomic analysis. Note: The fold difference in relative abundance of genes in BF and QQBF were treated using Exponential. **Figure S13.** Test results of the degradation of toluene by *Rhodococcus sp.* BH4. **Table S1.** Development of waste gas biofilter technology. **Table S2.** Composition of the nutrient solution. **Table S3.** The operating conditions of the two biofilters. **Table S4.** The results of Ergun equation fitting at the 25th day and the 65th day. **Table S5.** Statistic differences in EPS concentrations. **Table S6.** Quenching rate (k) of AHLs in sludge samples at different times. **Table S7.** The description and relative abundance of QS-related genes in BF and QQBF samples. **Table S8.** Topological properties of the empirical pMENs of the biofilm communities in BF and QQBF biofilms during the operation and their associated random pMENs. **Table S9.** Keystone species' centrality indexes of networks in biofilm communities sourced from BF and QQBF biofilms. **Table S10.** Taxonomic information and average abundance of keystone taxa observed in ecological networks in biofilm communities sourced from BF and QQBF biofilms. **Table S11.** Typical QS genes description and function in QS regulatory pathways in BF and QQBF biofilm samples based on metagenomic analysis. **Table S12.** Summary of the role of *Rhodococcus sp.* BH4 in bioreactors. **Supplementary Method S1.** The biofilm adhesion strength test. **Supplementary Method S2.** 16S rDNA sequencing. **Supplementary Method S3.** Metagenomics Sequencing Analysis. **Supplementary Method S4.** The calculation of biofilter performance and pressure drop curve fitting methods.

## Data Availability

Sequence data of all samples are available at the following: NCBI GenBank accession numbers: PRJNA818815 & PRJNA818845.
