# Supplementary material for: Microbial community regulation and performance enhancement in gas biofilters by interrupting bacterial communication
Source: Microbiome. 2022 Sep 19;10:150. doi: 10.1186/s40168-022-01345-5 (PMC9484056; doi:10.1186/s40168-022-01345-5)
Supplement: Supplementary file 2 — Additional file 1: Figure S1. The schematic diagram of biofilters setup. Figure S2. Schematic diagram of biofilm adhesion strength test. Figure S3. Effect of different dosage of Rhodococcus sp.BH4 on biofilm formation. Figure S4. (a) AHL quenching rate and (b) quorum quenching activity towards the exogenous AHL in the biofilm samples on Days 25, 45, 65 and 90. Figure S5. The sample distance matrix obtained based on the bray Curtis algorithm. The results were used to test whether the difference between groups is significantly greater than the difference within the group. Figure S6. Chao1 index, number of OTUs, Shannon index and pielou_e index in BF and QQBF biofilms. Figure S7. Relative abundance of biomarkers at the phylum level in (a) BF and (b) QQBF. The biomarkers were chosen through Kruskal-Wallis test. Figure S8. Comparison of relative abundance of QS-related bacteria in BF and QQBF biofilms at day 25 and 90. Figure S9. Comparison of gene expression levels in microbial samples at day 90 between BF and QQBF based on metagenomic analysis. Figure S10. Comparison of the number of up-regulated and down-regulated genes in microbial samples (BF vs QQBF) at day 90 of BF and QQBF based on metagenomic analysis. Figure S11. Differential gene enrichment results in microbial samples at day 90 of BF and QQBF based on KEGG database annotations. Figure S12. Up-regulation of QS genes in BF biofilm samples compared to QQBF in QS regulatory pathways based on metagenomic analysis. Note: The fold difference in relative abundance of genes in BF and QQBF were treated using Exponential. Figure S13. Test results of the degradation of toluene by Rhodococcus sp. BH4. Table S1. Development of waste gas biofilter technology. Table S2. Composition of the nutrient solution. Table S3. The operating conditions of the two biofilters. Table S4. The results of Ergun equation fitting at the 25th day and the 65th day. Table S5. Statistic differences in EPS concentrations. Table S6. Quenching rate ( [file 40168_2022_1345_MOESM1_ESM.docx]

**Supporting information for**

Microbial community regulation and performance enhancement in gas biofilters by interrupting bacteria communication

Yong-Chao Wang ^a,b^, Yu-Ting Lin ^a,b^, Can Wang ^a,b,^*, Zhen Tong ^a,b^, Xu-Rui Hu ^a,b^, Ya-Hui Lv ^a,b^, Guan-Yu Jiang ^a,b^, Meng-Fei Han ^a,b^, Ji-Guang Deng ^c^, Hsing-Cheng Hsi ^d^, Chung-Hak Lee ^e^

*^a^School of Environmental Science and Engineering, Tianjin University, Tianjin 300072, China*

*^b^Tianjin Key Lab of Indoor Air Environmental Quality Control, Tianjin 300072, China*

*^c^College of Environmental and Energy Engineering, Beijing University of Technology, Beijing 100124, China*

*^d^Graduate Institute of Environmental Engineering, National Taiwan University, No. 1, Sec. 4, Roosevelt Rd., Taipei 106, Taiwan*

*^e^* *School of Chemical and Biological Engineering, Seoul National University, Seoul 08826, Republic of Korea*

*Corresponding author:

Prof. Can Wang

School of Environmental Science and Engineering

Tianjin University

Yaguan Road#135, Tianjin, 300350, China

Tel/Fax: +86-22-27406057

E-mail: [wangcan@tju.edu.cn](mailto:wangcan@tju.edu.cn)

**Number of Pages: 43**

**Number of Figures: 13**

**Number of Tables: 12**

**Supplementary Method: Pages S6-S10**

**Supplementary Figure:** **Pages S11-S20**

**Figure S1.** The schematic diagram of biofilters setup.

**Figure S2.** Schematic diagram of biofilm adhesion strength test.

**Figure S3.** Effect of different dosage of *Rhodococcus sp*.BH4 on biofilm formation.

**Figure S4.** (a) AHL quenching rate and (b) quorum quenching activity towards the exogenous AHL in the biofilm samples on Days 25, 45, 65 and 90.

**Figure S5.** The sample distance matrix obtained based on the bray Curtis algorithm. The results were used to test whether the difference between groups is significantly greater than the difference within the group.

**Figure S6.** Chao1 index, number of OTUs, Shannon index and pielou_e index in BF and QQBF biofilms.

**Figure S7.** Relative abundance of biomarkers at the phylum level in (a) BF and (b) QQBF. The biomarkers were chosen through Kruskal-Wallis test.

**Figure S8.** Comparison of relative abundance of QS-related bacteria in BF and QQBF biofilms at day 25 and 90.

**Figure S9.** Comparison of gene expression levels in microbial samples at day 90 between BF and QQBF based on metagenomic analysis.

**Figure S10.** Comparison of the number of up-regulated and down-regulated genes in microbial samples (BF vs QQBF) at day 90 of BF and QQBF based on metagenomic analysis.

**Figure S11.** Differential gene enrichment results in microbial samples at day 90 of BF and QQBF based on KEGG database annotations.

**Figure S12.** Up-regulation of QS genes in BF biofilm samples compared to QQBF in QS regulatory pathways based on metagenomic analysis. Note: The fold difference in relative abundance of genes in BF and QQBF were treated using Exponential.

**Figure S13.** Test results of the degradation of toluene by *Rhodococcus sp.* BH4

**Supplementary Table: Pages S21-S37**

**Table S1.** Development of waste gas biofilter technology.

**Table S2**. Composition of the nutrient solution.

**Table S3**. The operating conditions of the two biofilters.

**Table S4.** The results of Ergun equation fitting at the 25th day and the 65th day.

**Table S5.** Statistic differences in EPS concentrations.

**Table S6.** Quenching rate (k) of AHLs in sludge samples at different times.

**Table S7**. The description and relative abundance of QS-related genes in BF and QQBF samples.

**Table S8**. Topological properties of the empirical pMENs of the biofilm communities in BF and QQBF biofilms during the operation and their associated random pMENs.

**Table S9**. Keystone species' centrality indexes of networks in biofilm communities sourced from BF and QQBF biofilms.

**Table S10.** Taxonomic information and average abundance of keystone taxa observed in ecological networks in biofilm communities sourced from BF and QQBF biofilms.

**Table S11**. Typical QS genes description and function in QS regulatory pathways in BF and QQBF biofilm samples based on metagenomic analysis.

**Table S12.** Summary of the role of *Rhodococcus sp.* BH4 in bioreactors

**Supplementary Method**

**Supplementary Method S1--The biofilm adhesion strength test.**

The activated sludge was collected from Tianjin Jinnan Waste-water Treatment Plant. *Rhodococcus* sp. BH4 was incubated for 24 h in an Erlenmeyer flask at 28 ℃ and 120 rpm in Luria-Bertani (LB) broth. Then, both activated sludge and *Rhodococcus* sp. BH4 were collected after centrifugation at 4000 rpm for 10 min, washed three times with buffered saline solution (0.9% NaCl, pH=7) and resuspended in buffered saline solution. The OD values were adjusted to 1.0 using a UV spectrophotometer (UV1100, Shanghai Mapada Instruments, China).

The effect of *Rhodococcus sp.* BH4 on the adhesion strength of the biofilm on the surface of the wood was tested using shear force of water flow. When microorganisms adhere onto the surface, the biofilm interacts with the surface of the carrier, allowing biofilm adhesion. The shear force of water flow affects biofilm adhesion. When the shear force is sufficiently large, the biofilm detaches. The shear stress can be calculated as in Eqs. S1–3 ^1, 2^:

| $\tau_{0}=\rho gRJ$ | $\left( S1 \right)$ |
| --- | --- |
| $J=cot\alpha$ | $\left( S2 \right)$ |
| $R=\frac{A}{\chi}$ | $\left( S3 \right)$ |

where τ_0_ is the shear stress (Pa); *ρ*, *g*, *R*, and *J* are the fluid density (kg/m^3^), acceleration of gravity (m/s^2^), hydraulic radius, and water surface slope, respectively; *A*, *χ*, and *α* are the cross-sectional area of water flow, the wetted perimeter, and the angle between the carrier and the vertical plane, respectively.

Under the action of continuous water-current shearing force, the biofilm shedding condition was observed. The test diagram is shown in FigureS2. The biofilm adhesion strength was determined by calculating the shear viscosity as in Eq. S4:

| $\eta=\tau t$ | $\left( S4 \right)$ |
| --- | --- |

where *η* is shear viscosity, *τ* (Pa) is shear stress, and *t* is the washing time.

Before the biofilm formation experiment, each board was put in ultrapure water for 15 minutes to make it saturated with water. In the subsequent experiments, repeated tests and blank boards were used as controls to eliminate the impact of water.

**Supplementary Method S2--16S rDNA sequencing**

The filler samples in the two biofilters were collected at each sampling point (the 25^th^, 45^th^, 65^th^, and 90^th^ day). The biofilm on the filler surface was stripped under the ultrasonic vibration condition. Then the biofilm samples were centrifuged at 8000 rpm for 5 min, repeated three times. DNA from different samples was extracted using the E.Z.N.A. ®Stool DNA Kit (D4015, Omega, Inc., USA) according to manufacturer’s instructions. The V3-V4 region of the bacteria 16S rRNA gene was amplified with primers 341F (5'-CCTACGGGNGGCWGCAG-3') and 805R (5'-GACTACHVGGGTATCTAATCC-3'). Then PCR reactions were conducted following a previous study and the PCR products were confirmed with 2% agarose gel electrophoresis. Afterwards, the AMPure XT beads (Beckman Coulter Genomics, Danvers, MA, USA) and Qubit (Invitrogen, USA) were used to purify and quantify the PCR products. Amplicons were then used for high-throughput sequencing on NovaSeq PE250 platform at LC-Bio Technology Co., Ltd, Hang Zhou, Zhejiang Province, China. Analysis of the relative abundance of QS-related genes in BF and QQBF by functional gene prediction.

**Supplementary Method S3--Metagenomics Sequencing Analysis**

DNA from different samples was extracted using The E.Z.N.A.® Stool DNA Kit (D4015-02, Omega, Inc., USA) according to manufacturer’s instructions. The reagent which was designed to uncover DNA from trace amounts of sample has been shown to be effective for the preparation of DNA of most bacteria. Sample blanks consisted of unused swabs processed through DNA extraction and tested to contain no DNA amplicons. The total DNA was eluted in 50 µl of Elution buffer by a modification of the procedure de-scribed by manufacturer (QIAGEN) and stored at -80°C until measurement in the PCR by LC-BIO TECHNOLOGIES (HANGZHOU) CO., LTD., Hang Zhou, Zhejiang Province, China.

DNA library was constructed by TruSeq Nano DNA LT Library Preparation Kit (FC-121-4001). DNA was fragmented by dsDNA Fragmentase (NEB, M0348S) by incubate at 37°C for 30min. Library construction begins with fragmented cDNA. Blunt-end DNA fragments are generated using a combination of fill-in reactions and exonuclease activity, and size selection is performed with provided sample purification beads. An A-base is then added to the blunt ends of each strand, preparing them for ligation to the indexed adapters.Each adapter contains a T-base overhang for ligating the adapter to the A-tailed fragmented DNA. These adapters contain the full complement of sequencing primer hybridization sites for single, paired-end, and indexed reads. Single- or dual-index adapters are ligated to the fragments and the ligated products are amplified with PCR by the following conditions: initial denaturation at 95oCfor 3 min; 8cycles of denaturation at 98 ℃ for 15 sec, annealing at 60oCfor 15 sec, and extension at 72 ^o^C for 30 sec; and then final extension at 72 ^o^C for 5 min.

Raw sequencing reads were processed to obtain valid reads for further analysis. First, sequencing adapters were removed from sequencing reads using cutadapt v1.9. Secondly, low quality reads were trimmed by fqtrim v0.94 using a sliding-window algorithm. Thirdly, reads were aligned to the host genome using bowtie2 v2.2.0 to remove host contamination. Once quality-filtered reads were obtained, they were de novo assembled to construct the metagenome for each sample by IDBA-UD v1.1.1. All coding regions (CDS) of metagenomic contigs were predicted by MetaGeneMark v3.26.

CDS sequences of all samples were clustered by CD-HIT v4.6.1 to obtain unigenes. Unigene abundance for a certain sample were estimated by TPM based on the number of aligned reads by bowtie2 v2.2.0. The lowest common ancestor taxonomy of unigenes were obtained by aligning them against the NCBI NR database by DIAMOND v 0.9.14. Similarly, the functional annotation (GO, KEGG, eggnog, CAZy, CARD, PHI, MGEs, VFDB) of unigenes were obtained. Based on the taxonomic and functional annotation of unigenes, along with the abundance profile of unigenes, the differential analysis was carried out at each taxonomic or functional or gene-wise level by Fisher's exact test (non-replicated groups) or Kruskal-Wallis test (replicated groups).

**Supplementary Method S4--The calculation of biofilter performance and pressure drop curve fitting methods**

Biomass inside the biofilters were intermittently measured on an electronic scale using the weighing method described by Hu et al. The removal efficiency (*RE*), biomass density (*B_t_*) and Elimination capacity (*EC*) were calculated using Eqs. S5–7:

| $RE=\frac{C_{in}-C_{out}}{C_{in}}\times100\%$ | (S5) |
| --- | --- |
| $B_{t}=\frac{m_{t}-m_{0}}{V}$ | (S6) |
| $EC=\frac{{(C}_{in}-C_{out})\times Q}{m_{t}-m_{0}}$ | (S7) |

where *C_in_* and *C_out_* (mg/m^3^) are the toluene concentrations at the inlet and outlet, respectively; *m_0_* and *m_t_* (kg) are the biofilter weights on the initial and sampling days, respectively; *V* (m^3^) is the filter bed volume; and *Q* (m^3^/h) is the air flow rate.

The relationship between the pressure drop values and the flow rate was analyzed using Ergun equation (Eq. S8):

| $\Delta P=\alpha v^{2}+\beta v$ | (S8) |
| --- | --- |

where $\Delta P$ is the pressure drop (Pa m^-1^), *v* is the gas velocity (m s^-1^), and *α* (Pa h^-2^ m^-3^) and β (Pa h^-1^ m^-2^) are the linear regression parameters.

**Supplementary Figure**


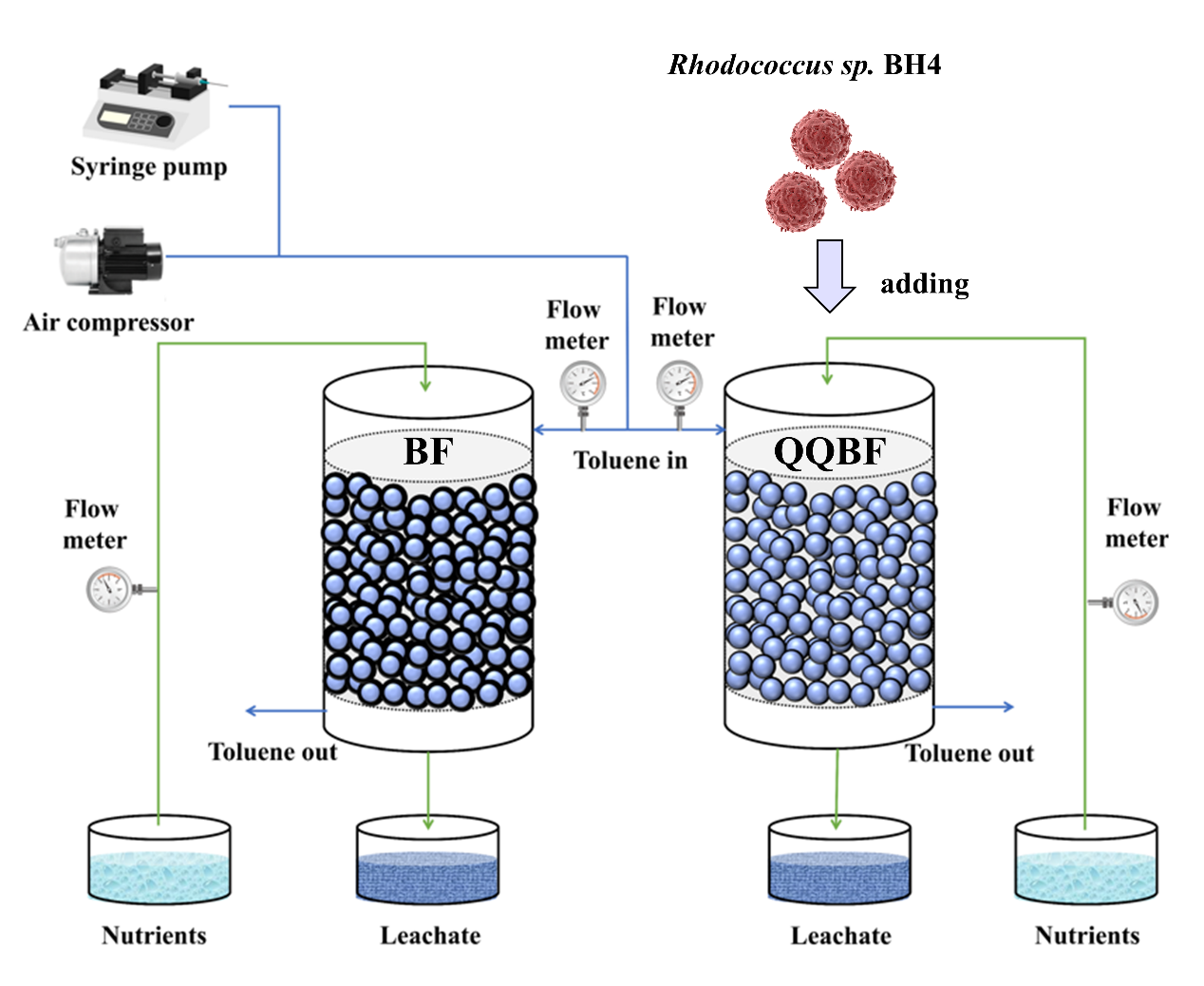


**Figure S1.** The schematic diagram of biofilters setup.


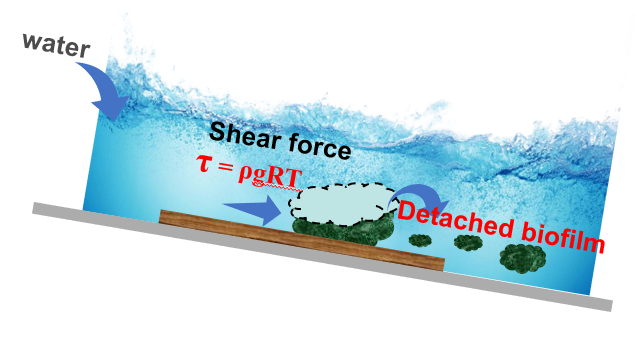


**Figure S2.** Schematic diagram of biofilm adhesion strength test.


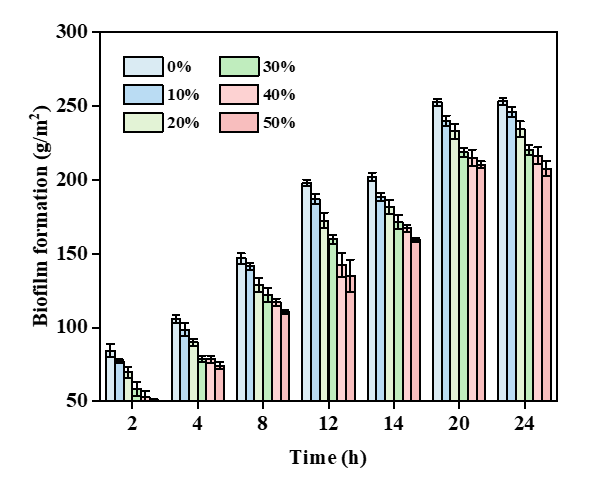


**Figure S3.** Effect of different dosage of *Rhodococcus sp*.BH4 on biofilm formation.


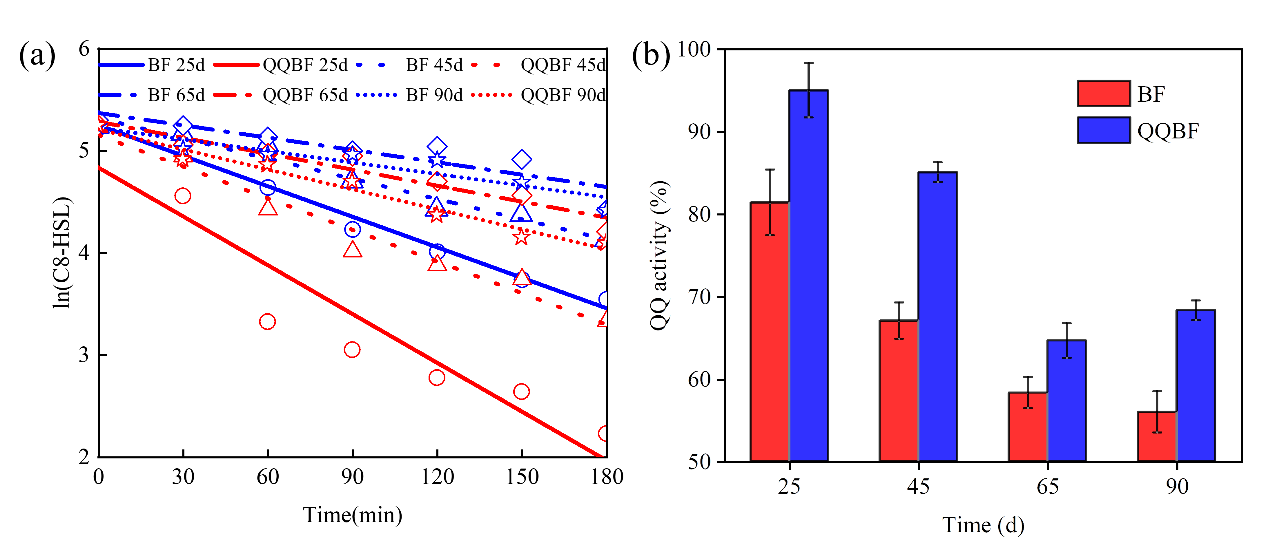


**Figure S4.** (a) AHL quenching rate and (b) quorum quenching activity towards the exogenous AHL in the biofilm samples on Days 25, 45, 65 and 90.


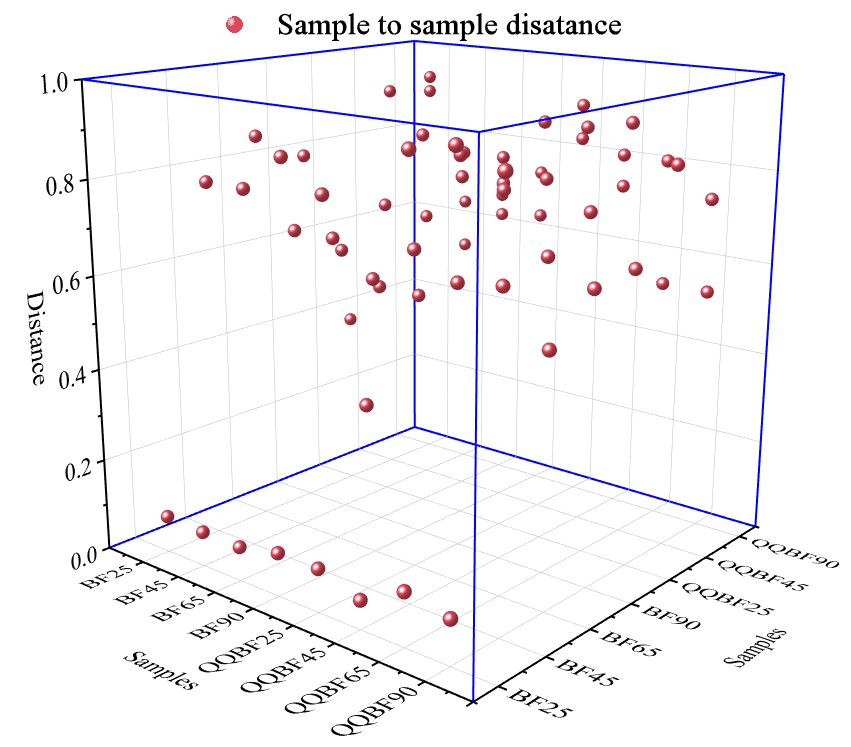


**Figure S5.** The sample distance matrix obtained based on the bray Curtis algorithm. The results were used to test whether the difference between groups is significantly greater than the difference within the group.


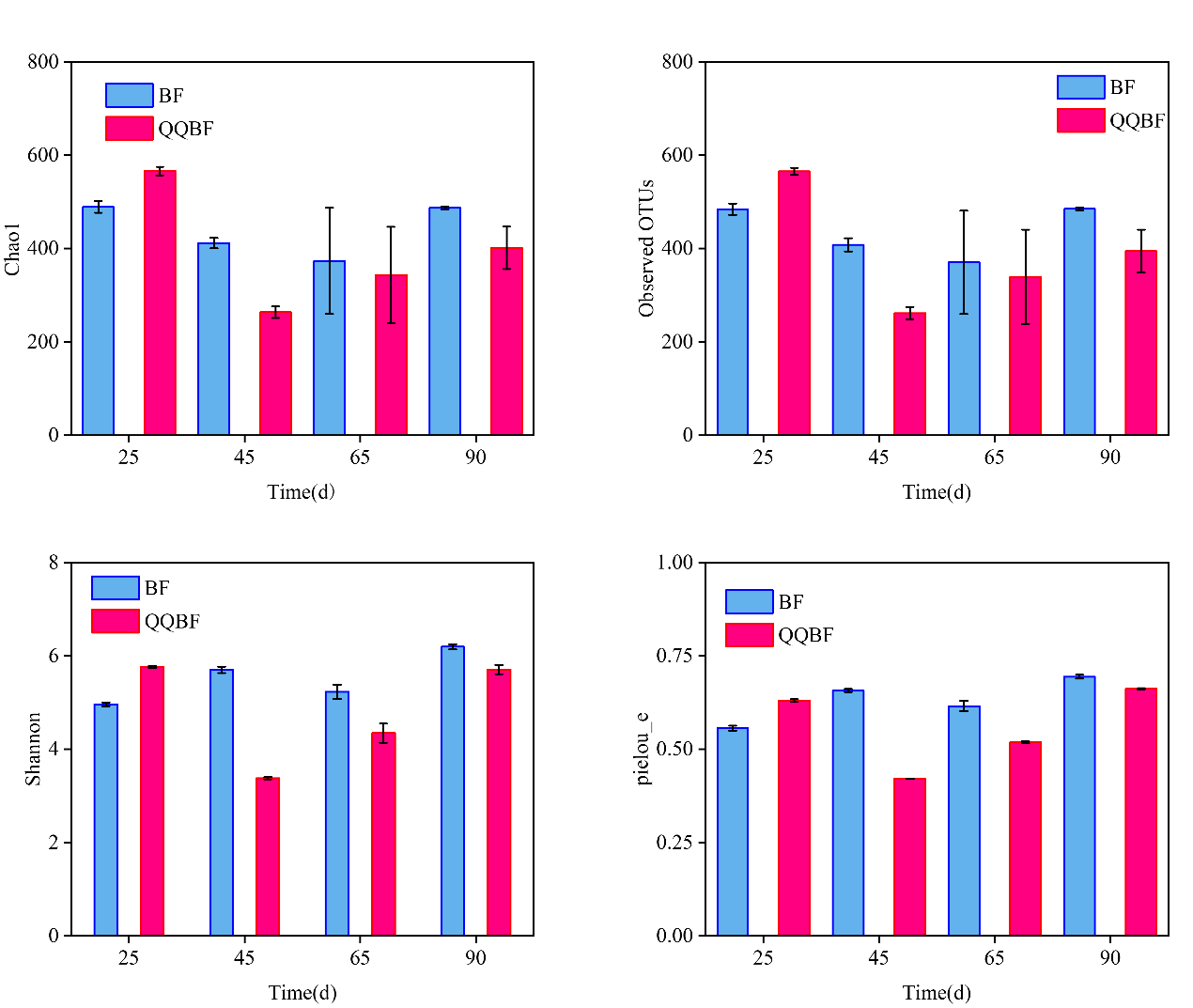


**Figure S6.** Chao1 index, number of OTUs, Shannon index and pieloue index in BF and QQBF biofilms.


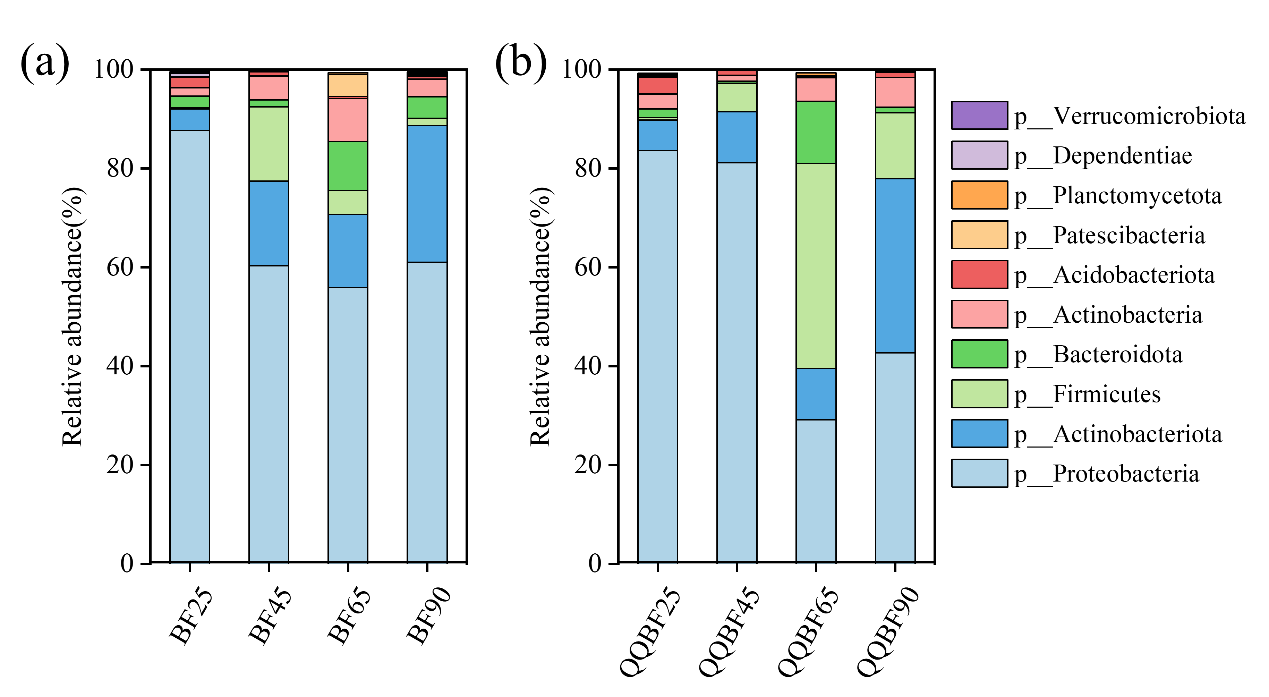


**Figure S7.** Relative abundance of biomarkers at the phylum level in (a) BF and (b) QQBF. The biomarkers were chosen through Kruskal-Wallis test.


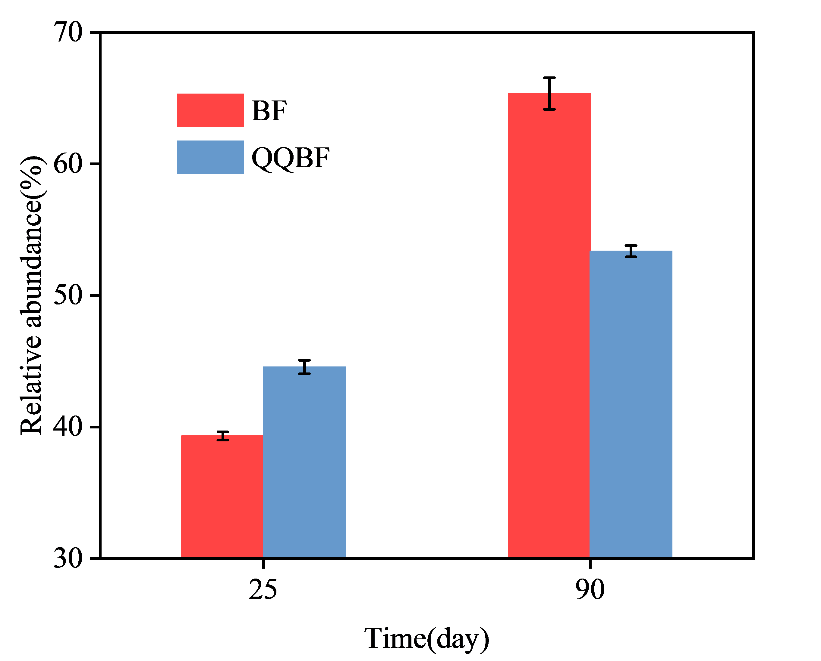


**Figure S8.** Comparison of relative abundance of QS-related bacteria in BF and QQBF biofilms at day 25 and 90.


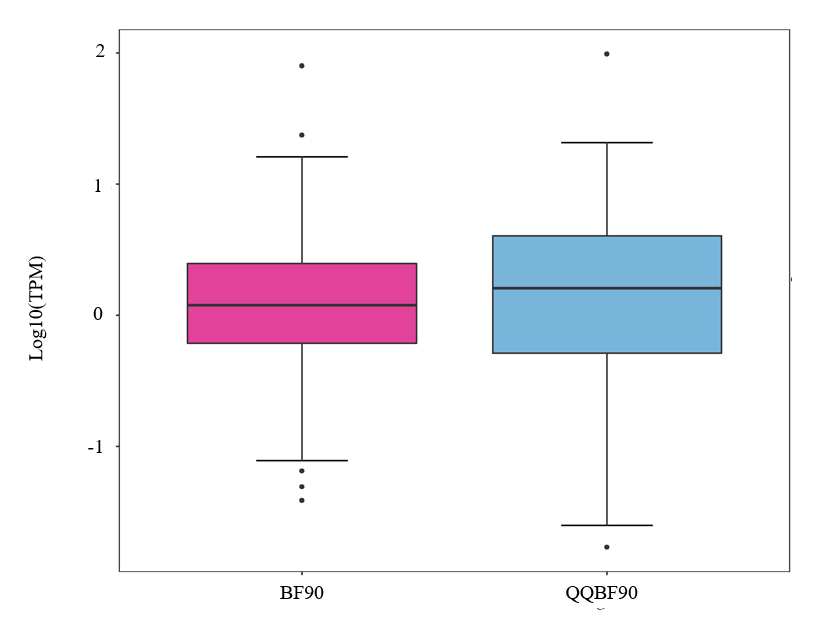


**Figure S9*.*** Comparison of gene expression levels in microbial samples at day 90 between BF and QQBF based on metagenomic analysis.


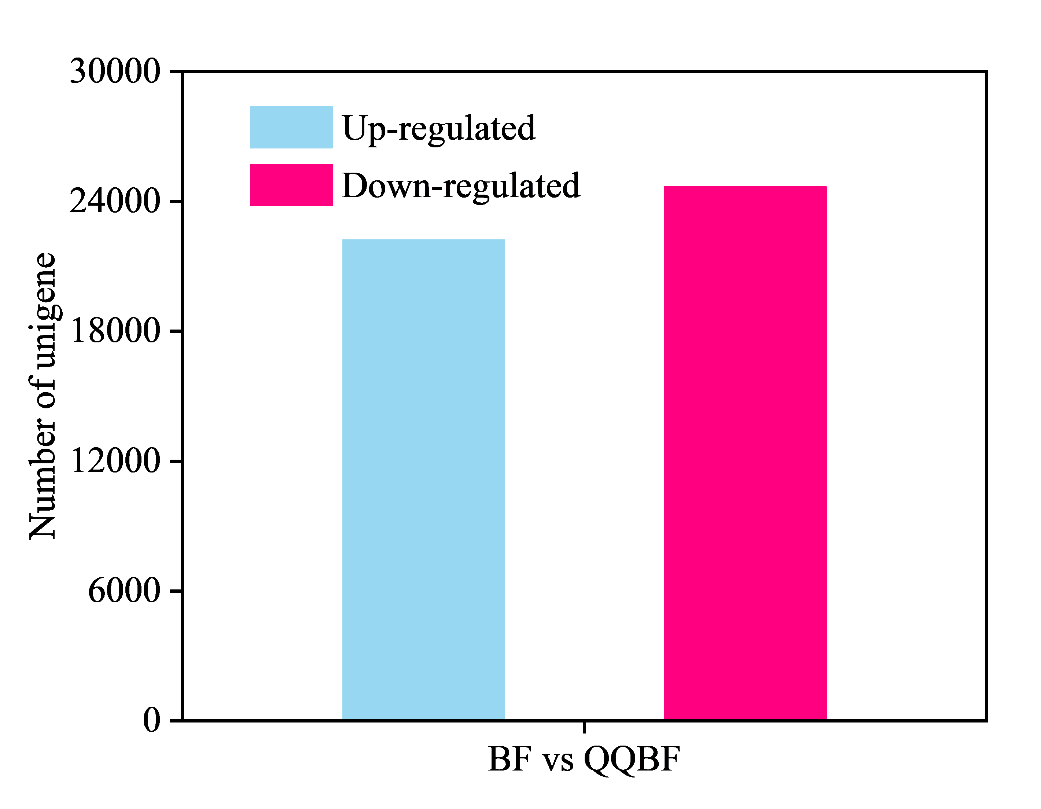


**Figure S10.** Comparison of the number of up-regulated and down-regulated genes in microbial samples (BF vs QQBF) at day 90 of BF and QQBF based on metagenomic analysis.


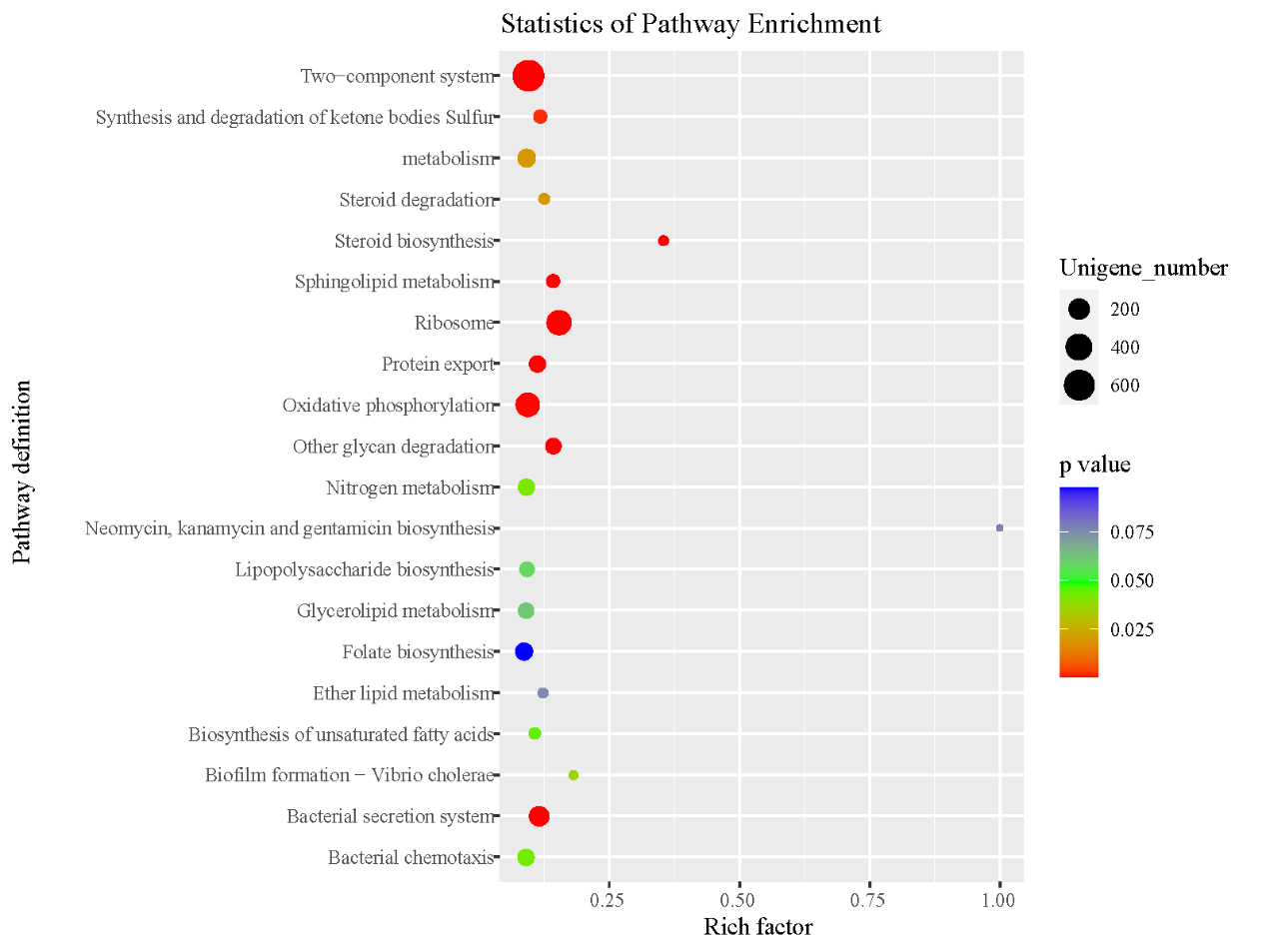


**Figure S11.** Differential gene enrichment results in microbial samples at day 90 of BF and QQBF based on KEGG database annotations.


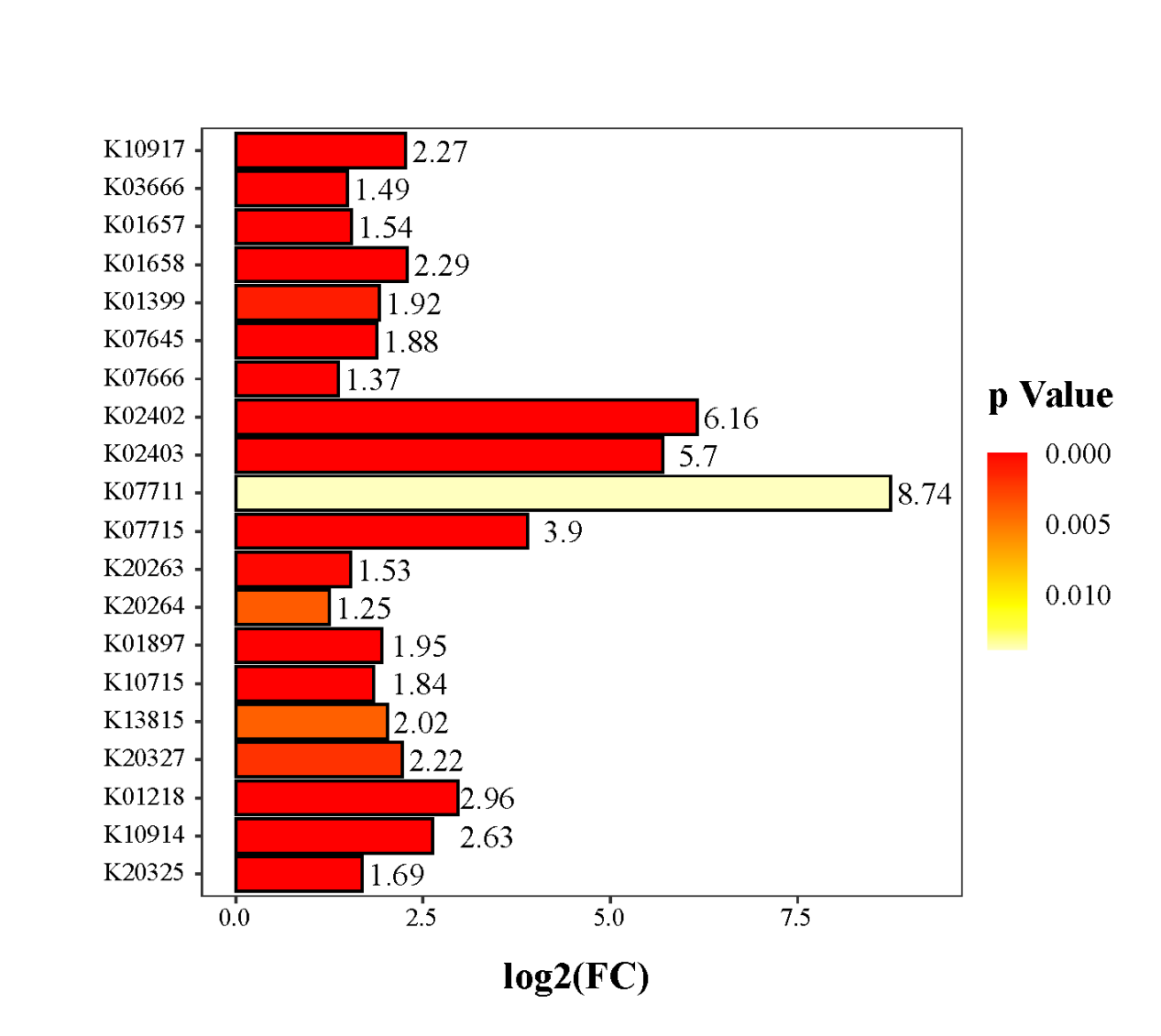


**Figure S12.** Up-regulation of QS genes in BF biofilm samples compared with QQBF in QS regulatory pathways based on metagenomic analysis. Note: The fold difference in relative abundance of genes in BF and QQBF were treated using Exponential.


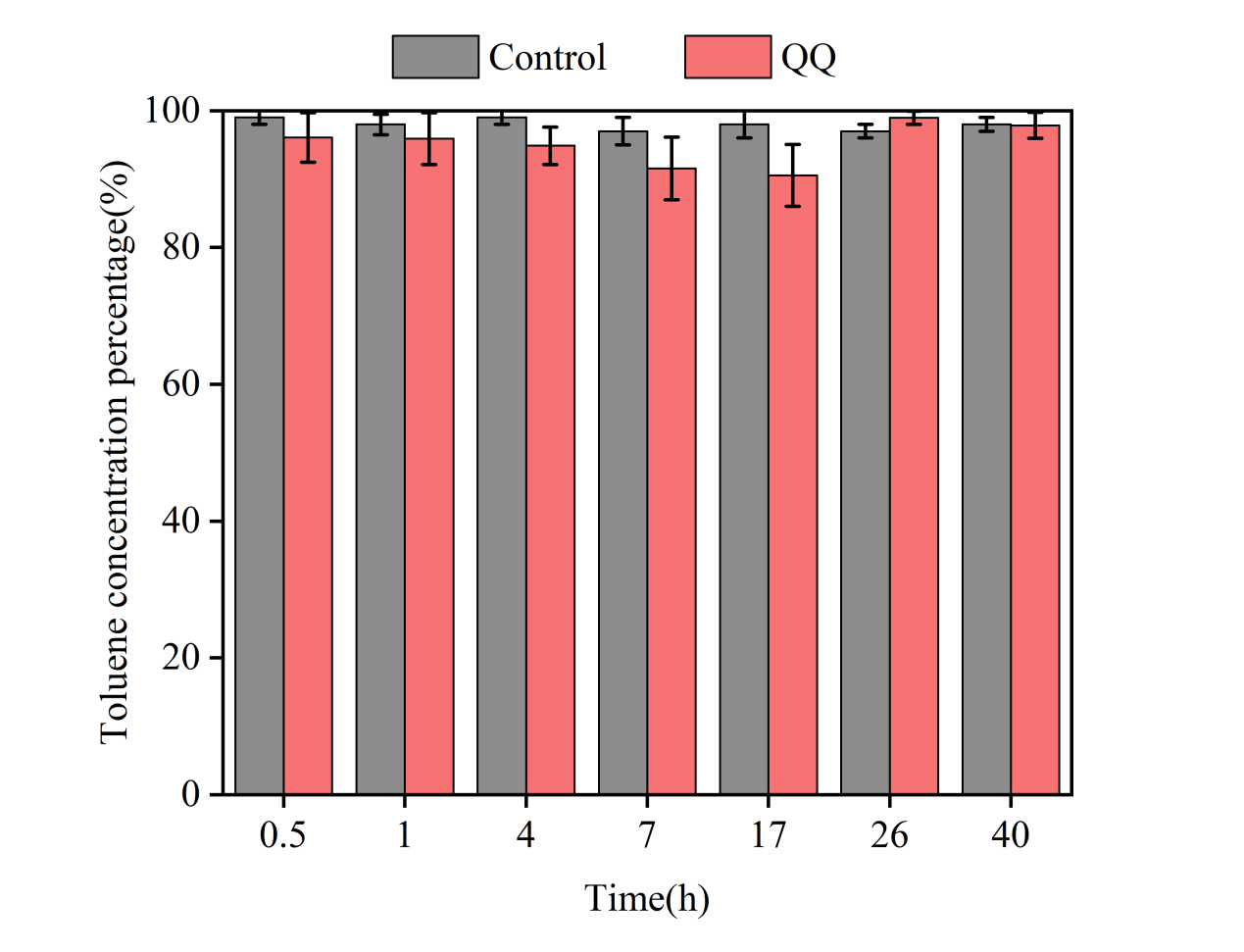


**Figure S13.** Test results of the degradation of toluene by *Rhodococcus sp.* BH4

**Supplementary Table**

**Table S1. Development of waste gas biofilter technology.**

| Reactor type | Time | Target | Medium | Reference |
| --- | --- | --- | --- | --- |
| Soil Biofilter | 1950s | Odors, H_2_S | Soil microorganisms | ^3^ |
| Biofilter | 1960s | Odors from a variety of sources | Compost derived from municipal solid waste | ^3^ |
| Biofilter | 1960-1970s | Odors; VOCs | Air distribution system | ^3^ |
| Biofilter | Early 1980s | VOCs and air toxics | Compost derived from municipal waste, bark, tree trimmings and leaves | ^3^ |
| Biofilter | Early 1990s | Phenol | Peat and glass beads | ^4^ |
| Biofilter | 1990s | Butyl acetate and styrene | Compost and small balls such as plastic balls or silicone balls | ^5^ |
| Biofilter | 1990s | VOCs like benzene, toluene, ethylbenzene and xylene | Compost and activated carbon | ^6^ |
| Biofilter | Early 20th century | VOCs like butyl acetate, methanol, acetone and toluene | Peat | ^7^ |
| Biofilter | Early 20th century | Toluene | Perlite | ^8^ |
| Biofilter | 2000s | Chlorobenzene | Ordinary filler with ozone | ^9^ |
| Bio-trickling filter | 2000s | Hydrophilic gases produced by livestock farms | Bio-trickling filters | ^10^ |
| Bio-trickling filter | 2010s | VOCs like toluene | Bio-trickling filters filled with conductive packing | ^11^ |
| Biofilter | 2015 | Mixed high temperature VOCs | Perlite | ^12^ |
| Two-Liquid Phase Biofilter | 2016 | VOCs like dichloromethane | Perlite and ceramsite | ^13^ |
| Biofilter | 2020 | Chlorinated VOCs like chlorobenzene | Ordinary filler added CaCO_3_ and 3D matrix material | ^13^ |
| Combined photodegradation and biofiltration | 2020 | Various VOCs | Ordinary filler | ^13^ |
| Biofilter | 2022 | VOCs like methanol | Ordinary filler added quorum quenching enzyme | ^14^ |

**Table S2**. Composition of the nutrient solution.

| Ingredients | Concentration (g/L) |
| --- | --- |
| Na_2_HPO_4_·12H_2_O | 7 |
| KH_2_PO_4_ | 2 |
| (NH_4_)_2_SO_4_ | 2.5 |
| MgSO_4_·7H_2_O | 0.2 |
| H_3_BO_3_ | 0.003 |
| Na_2_MoO_4_·H_2_O | 0.004 |
| Ca(NO_3_)_2_·4H_2_O | 0.6 |
| CuSO_4_·5H_2_O | 0.04 |
| FeSO_4_·7H_2_O | 0.2 |
| ZnSO_4_·7H_2_O | 0.02 |
| MnSO_4_·4H_2_O | 0.02 |

**Table S3**. The operating conditions of the two biofilters.

|  | Period of operation (day) | 0-120 |
| --- | --- | --- |
| BF | Inlet toluene concentration (mg m^-3^) | 400±100 |
|  | EBRT^*^ of the biofilter (s) | 33 |
|  | Flow rate (m^-3^ h) | 0.1 |
|  | Spray frequency and volume | Nutrient: 100ml/8h |
|  | Composition of initial film sludge | Activated sludge after domestication |
| QQBF | Inlet toluene concentration (mg m^-3^) | 400±100 |
|  | EBRT of the biofilter (s) | 33 |
|  | Flow rate (m^-3^ h) | 0.1 |
|  | Spray frequency and volume | Nutrient: 100ml/8h |
|  | Composition of initial film sludge | 70% activated sludge after domestication and 30% *Rhodococcus* sp. BH4 solution |

EBRT*: empty bed residence time.

**Table S4.** The results of Ergun equation fitting at the 25th day and the 65th day.

|  | 25th day | | 65th day | |
| --- | --- | --- | --- | --- |
|  | Fitting formula | R-squared | Fitting formula | R-squared |
| BF | y=0.36*v*^2^+0.003*v* | 0.9975 | y=0.63*v*^2^+0.006*v* | 0.9942 |
| QQBF | y=0.26*v*^2^+0.007*v* | 0.9957 | y=0.29*v*^2^+0.005*v* | 0.9989 |

**Table S5.** Statistic differences in EPS concentrations.

| **Day** | **Protein concentration** | **Polysaccharide concentration** |
| --- | --- | --- |
| **25** | BF>QQBF; p=0.0025 | BF>QQBF; p=0.003 |
| **45** | BF>QQBF; p=0.013 | BF>QQBF; p=0.042 |
| **65** | BF>QQBF; p=0.0008 | BF>QQBF; p=0.0032 |
| **90** | BF>QQBF; p=0.0025 | BF>QQBF; p=0.037 |

**Table S6.** Quenching rate (k; min^-1^) of AHLs in sludge samples at different times.

| **Day** | **25** | **45** | **65** | **90** |
| --- | --- | --- | --- | --- |
| **BF** | 0.00995 | 0.00661 | 0.00402 | 0.00378 |
| **QQBF** | 0.01592 | 0.01031 | 0.00521 | 0.00651 |

**Table S7**. The description and relative abundance of QS-related genes in BF and QQBF samples.

| **KOEntry** | **description** | **BF25 VS QQBF25;** | **BF25 VS QQBF90;** |
| --- | --- | --- | --- |
| **K01897** | ACSL, fadD; long-chain acyl-CoA synthetase [EC:6.2.1.3] | __ | __ |
| **K03217** | yidC, spoIIIJ, OXA1, ccfA; YidC/Oxa1 family membrane protein insertase | QQBF25> BF25; p<0.01 | __ |
| **K01626** | E2.5.1.54, aroF, aroG, aroH; 3-deoxy-7-phosphoheptulonate synthase [EC:2.5.1.54] | __ | __ |
| **K02055** | ABC.SP. S; putative spermidine/putrescine transport system substrate-binding protein | __ | __ |
| **K02053** | ABC.SP. P; putative spermidine/putrescine transport system permease protein | __ | __ |
| **K02052** | ABC.SP. A; putative spermidine/putrescine transport system ATP-binding protein | BF25> QQBF25; p<0.01 | __ |
| **K18139** | oprM, emhC, ttgC, cusC, adeK, smeF, mtrE, cmeC, gesC; outer membrane protein, multidrug efflux system | BF25> QQBF25; p<0.01 | __ |
| **K01999** | livK; branched-chain amino acid transport system substrate-binding protein | QQBF25> BF25; p<0.01 | __ |
| **K03666** | hfq; host factor-I protein | __ | __ |
| **K03110** | ftsY; fused signal recognition particle receptor | QQBF25> BF25; p<0.01 | __ |
| **K09823** | zur; Fur family transcriptional regulator, zinc uptake regulator | __ | BF90> QQBF90; p<0.01 |
| **K01657** | trpE; anthranilate synthase component I [EC:4.1.3.27] | __ | __ |
| **K02032** | ABC.PE.A1; peptide/nickel transport system ATP-binding protein | __ | __ |
| **K01998** | livM; branched-chain amino acid transport system permease protein | __ | __ |
| **K07645** | qseC; two-component system, OmpR family, sensor histidine kinase QseC [EC:2.7.13.3] | __ | BF90> QQBF90; p<0.01 |
| **K01580** | E4.1.1.15, gadB, gadA, GAD; glutamate decarboxylase [EC:4.1.1.15] | __ | QQBF90> BF90; p<0.01 |
| **K06998** | phzF; trans-2,3-dihydro-3-hydroxyanthranilate isomerase [EC:5.3.3.17] | __ | __ |
| **K07711** | glrK, qseE; two-component system, NtrC family, sensor histidine kinase GlrK [EC:2.7.13.3] | __ | __ |
| **K07715** | glrR, qseF; two-component system, NtrC family, response regulator GlrR | __ | __ |
| **K03070** | secA; preprotein translocase subunit SecA | QQBF25> BF25; p<0.01 | __ |
| **K02034** | ABC.PE.P1; peptide/nickel transport system permease protein | __ | __ |
| **K03076** | secY; preprotein translocase subunit SecY | QQBF25> BF25; p<0.01 | __ |
| **K03210** | yajC; preprotein translocase subunit YajC | QQBF25> BF25; p<0.01 | __ |
| **K03073** | secE; preprotein translocase subunit SecE | QQBF25> BF25; p<0.01 | __ |
| **K01114** | plc; phospholipase C [EC:3.1.4.3] | __ | __ |
| **K03071** | secB; preprotein translocase subunit SecB | __ | BF90> QQBF90; p<0.01 |
| **K02035** | ABC.PE.S; peptide/nickel transport system substrate-binding protein | __ | __ |
| **K02033** | ABC.PE.P; peptide/nickel transport system permease protein | __ | __ |
| **K02031** | ABC.PE.A; peptide/nickel transport system ATP-binding protein | __ | __ |
| **K07666** | qseB; two-component system, OmpR family, response regulator QseB | BF25> QQBF25; p<0.01 | BF90> QQBF90; p<0.01 |
| **K07344** | trbL; type IV secretion system protein TrbL | __ | __ |
| **K01497** | ribA, RIB1; GTP cyclohydrolase II [EC:3.5.4.25] | __ | BF90> QQBF90; p<0.01 |
| **K03075** | secG; preprotein translocase subunit SecG | QQBF25> BF25; p<0.01 | __ |
| **K01997** | livH; branched-chain amino acid transport system permease protein | __ | __ |
| **K01996** | livF; branched-chain amino acid transport system ATP-binding protein | __ | __ |
| **K01995** | livG; branched-chain amino acid transport system ATP-binding protein | __ | __ |
| **K10914** | crp; CRP/FNR family transcriptional regulator, cyclic AMP receptor protein | __ | __ |
| **K12990** | rfbF, rhlC; rhamnosyltransferase [EC:2.4.1.-] | BF25> QQBF25; p<0.01 | __ |
| **K13075** | ahlD, aiiA, attM, blcC; N-acyl homoserine lactone hydrolase [EC:3.1.1.81] | QQBF25> BF25; p<0.01 | __ |
| **K07692** | degU; two-component system, NarL family, response regulator DegU | __ | __ |
| **K03106** | SRP54, ffh; signal recognition particle subunit SRP54 [EC:3.6.5.4] | QQBF25> BF25; p<0.01 | __ |
| **K14051** | gmr; c-di-GMP phosphodiesterase Gmr [EC:3.1.4.52] | __ | __ |
| **K01658** | trpG; anthranilate synthase component II [EC:4.1.3.27] | __ | BF90> QQBF90; p<0.01 |
| **K01218** | gmuG; mannan endo-1,4-beta-mannosidase [EC:3.2.1.78] | __ | __ |
| **K10715** | rpfC; two-component system, sensor histidine kinase RpfC [EC:2.7.13.3] | QQBF25> BF25; p<0.01 | __ |
| **K10823** | oppF; oligopeptide transport system ATP-binding protein | __ | __ |
| **K02402** | flhC; flagellar transcriptional activator FlhC | BF25> QQBF25; p<0.01 | BF90> QQBF90; p<0.01 |
| **K02403** | flhD; flagellar transcriptional activator FlhD | BF25> QQBF25; p<0.01 | BF90> QQBF90; p<0.01 |
| **K11752** | ribD; diaminohydroxyphosphoribosylaminopyrimidine deaminase / 5-amino-6-(5-phosphoribosylamino) uracil reductase [EC:3.5.4.26 1.1.1.193] | QQBF25> BF25; p<0.01 | __ |
| **K15582** | oppC; oligopeptide transport system permease protein | __ | __ |
| **K14645** | K14645; serine protease [EC:3.4.21.-] | __ | BF90> QQBF90; p<0.01 |
| **K13815** | rpfG; two-component system, response regulator RpfG | __ | __ |
| **K15580** | oppA, mppA; oligopeptide transport system substrate-binding protein | __ | __ |
| **K18306** | mexH; membrane fusion protein, multidrug efflux system | __ | __ |
| **K07667** | kdpE; two-component system, OmpR family, KDP operon response regulator KdpE | QQBF25> BF25; p<0.01 | __ |
| **K12257** | secDF; SecD/SecF fusion protein | QQBF25> BF25; p<0.01 | __ |
| **K15583** | oppD; oligopeptide transport system ATP-binding protein | __ | __ |
| **K18098** | bjaR1, rpaR, rhiR; LuxR family transcriptional regulator, quorum-sensing system regulator BjaR1 | __ | __ |

**Table S8**. Topological properties of the empirical pMENs of the biofilm communities in BF and QQBF biofilms during the operation and their associated random pMENs.

| **Biofilm**  **community** | **Empirical networks** | | | | | | | **Random network** | | | |
| --- | --- | --- | --- | --- | --- | --- | --- | --- | --- | --- | --- |
|  | Network  size (n) | Similarity  threshold  (St) | R^2^ of  power  law | Average  connectivity  (avgK) | Harmonic  geodesic  distance  (HD) | Average  clustering  coefficient  (avgCC) | Modularity  (no. of  modules) | Transitivity  (Trans) | Average  σ (avgCC) | Harmonic  geodesic  distance  (HD±SD) | Average  clustering  coefficient  (avgCC±SD) |
| **BF** | 912 | 0.97 | 0.647 | 9.651 | 2.931 | 0.476 | 0.593 | 0.549 | 4.61 | 2.383±0.015 | 0.127±0.011 |
| **QQBF** | 705 | 0.97 | 0.545 | 10.846 | 2.729 | 0.518 | 0.458 | 0.554 | 3.07 | 2.184±0.016 | 0.211±0.016 |

The small-world coefficient σ = (CC/CCr)/(HD/HDr),

**Table S9**. Keystone species' centrality indexes of networks in biofilm communities sourced from BF and QQBF biofilms.

| Network | Name | Topological position | node.  degree | node.  betw | node.  stress | node.  evcent | Clustering.  Coefficient | No. module | Zi | Pi |
| --- | --- | --- | --- | --- | --- | --- | --- | --- | --- | --- |
| BF | OTU21 | Module hub | 24 | 1264.48 | 10045 | 0.08 | 0.30 | 2 | 2.60 | 0.41 |
| QQBF | OTU25 | Connector | 10 | 1107.77 | 23247 | 0.017 | 0.35 | 0 | 0.25 | 0.70 |
|  | OTU33 | Connector | 12 | 180.82 | 4360 | 0.025 | 0.44 | 2 | 0.18 | 0.71 |

**Table S10.** Taxonomic information and average abundance of keystone taxa observed in ecological networks in biofilm communities sourced from BF and QQBF biofilms.

| Network | OTU | Lowest taxonomic rank | Affiliate Phylum | Average abundance  (%) |
| --- | --- | --- | --- | --- |
| BF | OTU21 | *f__Microbacteriaceae* | *p__Actinobacteriota* | 0.34 |
| QQBF | OTU25 | *g__Gryllotalpicola* | *p__Actinobacteriota* | 0.25 |
|  | OTU33 | *g__Chujaibacter* | *p__Proteobacteria* | 0.22 |

**Table S11.** Typical QS genes description and function in QS regulatory pathways in BF and QQBF biofilm samples based on metagenomic analysis.

| **Gene name** | **KOEntry** | **Description** | **Statistic differences between BF and QQBF (P value)** | **Reference** |
| --- | --- | --- | --- | --- |
| **aphA** | K10917 | PadR family transcriptional regulator, regulatory protein AphA | BF>QQBF; *p*<0.01 | ^15, 16^ |
| **hfq** | K03666 | host factor-I protein | BF>QQBF; *p*<0.01 | ^17^ |
| **trpE** | K01657 | anthranilate synthase component I [EC:4.1.3.27] | BF>QQBF; *p*<0.01 | ^18^ |
| **trpG** | K01658 | anthranilate synthase component II [EC:4.1.3.27] | BF>QQBF; *p*<0.01 | ^18^ |
| **lasB** | K01399 | pseudolysin [EC:3.4.24.26] | BF>QQBF; *p*<0.01 | ^19, 20^ |
| **qseC** | K07645 | two-component system, OmpR family, sensor histidine kinase QseC [EC:2.7.13.3] | BF>QQBF; *p*<0.01 | ^21^ |
| **qseB** | K07666 | two-component system, OmpR family, response regulator QseB | BF>QQBF; *p*<0.01 | ^21^ |
| **flhC** | K02402 | flagellar transcriptional activator FlhC | BF>QQBF; *p*<0.01 | ^22^ |
| **flhD** | K02403 | flagellar transcriptional activator FlhD | BF>QQBF; *p*<0.01 | ^22^ |
| **glrK, qseE** | K07711 | two-component system, NtrC family, sensor histidine kinase GlrK [EC:2.7.13.3] | BF>QQBF; *p*<0.01 | ^23, 24^ |
| **glrR, qseF** | K07715 | two-component system, NtrC family, response regulator GlrR | BF>QQBF; *p*<0.01 | ^23, 24^ |
| **fusK** | K20263 | two-component system, NarL family, sensor histidine kinase FusK [EC:2.7.13.3] | BF>QQBF; *p*<0.01 | ^25^ |
| **fusR** | K20264 | two-component system, NarL family, response regulator FusR | BF>QQBF; *p*<0.01 | ^25^ |
| **ACSL, fadD** | K01897 | long-chain acyl-CoA synthetase [EC:6.2.1.3] | BF>QQBF; *p*<0.01 | ^26^ |
| **rpfC** | K10715 | two-component system, sensor histidine kinase RpfC [EC:2.7.13.3] | BF>QQBF; *p*<0.01 | ^27-29^ |
| **rpfG** | K13815 | two-component system, response regulator RpfG | BF>QQBF; *p*<0.01 | ^27-29^ |
| **xagB** | K20327 | glycosyltransferase XagB | BF>QQBF; *p*<0.01 | ^30^ |
| **gmuG** | K01218 | mannan endo-1,4-beta-mannosidase [EC:3.2.1.78] | BF>QQBF; *p*<0.01 | ^31^ |
| **crp** | K10914 | CRP/FNR family transcriptional regulator, cyclic AMP receptor protein | BF>QQBF; *p*<0.01 | ^32, 33^ |
| **fhrR** | K20325 | TetR/AcrR family transcriptional regulator, Clp-modulated transcription factor | BF>QQBF; *p*<0.01 | ^34^ |

**Table S12.** Summary of the role of *Rhodococcus sp.* BH4 in bioreactors

| **Types of bioreactor** | ***Rhodococcus sp.* BH4 adding method** | **Result** | **References** |
| --- | --- | --- | --- |
| **MBR** | QQ-sheets; QQ-beads;  5 mg/g media | QQ sheets exhibited 2.5-fold higher QQ activity and better washing effects than QQ-beads; TMP was reduced by 50%-80%. | ^35^ |
| **MBR** | QQ- Rotary microbial carrier frame; 25 mg/L | With QQ bacteria, the TMP increasing rate was decreased with 65% efficiency due to effect of physical shearing and QQ activity | ^36^ |
| **MBR** | QQ-cylinder; 7.5–11.4 mg/g media | Higher QQ activity and physical scouring by QQ-cylinder than QQ-beads due to the higher washing and QQ activity provided by higher surface area; TMP was reduced by 84%. | ^37^ |
| **MBR** | QQ-vessel | The QQ–vessel helped to operate MBR at lowest aeration intensity and reduced membrane biofouling. | ^38^ |
| **MBR** | QQ –beads; 6 mg/g media | In MBR provided with QQ-beads, the time for the TMP rise-up of 70 kPa was 10 times longer than control MBR. | ^39^ |
| **MBR** | QQ-vessel; 450 mg/L | The use of QQ-vessel in MBR substantially delayed the TMP rise-up without affecting treatment performance; TMP was reduced by 50%-70%. | ^40^ |
| **MBR** | QQ-vessel; 128.6 mg/L | The use of QQ-vessel in MBR substantially delayed the TMP rise-up without affecting treatment performance. | ^29^ |
| **MBR** | QQ-vessel; 167 mg/L | The degradation products by strain BH4 confirmed that AHL activity was inhibited. TMP was reduced by 60%. | ^29^ |
| **BF** | Dissociative QQ bacterial; 30% m/m | The biomass accumulation was reduced by 45%, the pressure drop was reduced by 70%, which improved a stable performance. | This study |

**MBR:** Membrane Bioreactor; **BF**: Biofilter.

**References**

1. Petit, F., The evaluation of grain shear stress from experiments in a pebble‐bedded flume. *Earth Surface Processes and Landforms* **1989,** *14*, (6), 499-508.

2. Berlamont, J. E.; Trouw, K.; Luyckx, G., Shear stress distribution in partially filled pipes. *Journal of Hydraulic Engineering* **2003,** *129*, (9), 697-705.

3. Leson, G.; Winer, A. M., Biofiltration: an innovative air pollution control technology for VOC emissions. *J Air Waste Manage* **1991,** *41*, (8), 1045-1054.

4. Abumaizar, R. J.; Kocher, W.; Smith, E. H., Biofiltration of BTEX contaminated air streams using compost-activated carbon filter media. *J Hazard Mater* **1998,** *60*, (2), 111-126.

5. Lu, C.; Chang, K., Biofiltration of butyl acetate and xylene mixtures using a trickle‐bed air biofilter. *Engineering in life sciences* **2004,** *4*, (2), 131-137.

6. Zilli, M.; Fabiano, B.; Ferraiolo, A.; Converti, A., Macro‐kinetic investigation on phenol uptake from air by biofiltration: Influence of superficial gas flow rate and inlet pollutant concentration. *Biotechnology and bioengineering* **1996,** *49*, (4), 391-398.

7. Aizpuru, A.; Malhautier, L.; Roux, J.-C.; Fanlo, J.-L., Biofiltration of a mixture of volatile organic emissions. *J Air Waste Manage* **2001,** *51*, (12), 1662-1670.

8. Van Groenestijn, J.; Van Heiningen, W.; Kraakman, N., Biofilters based on the action of fungi. *Water Science and Technology* **2001,** *44*, (9), 227-232.

9. Wang, C.; Xi, J. Y.; Hu, H. Y.; Yao, Y., Stimulative effects of ozone on a biofilter treating gaseous chlorobenzene. *Environmental ence & Technology* **2009,** *43*, (24), 9407-9412.

10. Delhoménie, M.-C.; Heitz, M., Biofiltration of air: a review. *Critical reviews in biotechnology* **2005,** *25*, (1-2), 53-72.

11. Wu, H.; Guo, C.; Yin, Z.; Quan, Y.; Yin, C., Performance and bacterial diversity of biotrickling filters filled with conductive packing material for the treatment of toluene. *Bioresource technology* **2018,** *257*, 201-209.

12. Hu, Q.-y.; Wang, C., Interaction of gaseous aromatic and aliphatic compounds in thermophilic biofilters. *J Hazard Mater* **2015,** *300*, 210-217.

13. Han, M.-F.; Wang, C.; Liu, H., Comparison of physical technologies for biomass control in biofilters treating gaseous toluene. *J Air Waste Manage* **2018,** *68*, (10), 1118-1125.

14. Wang, Y.-C.; Wang, C.; Han, M.-F.; Tong, Z.; Lin, Y.-T.; Hu, X.-R.; Deng, J.-G.; Hsi, H.-C., Inhibiting effect of quorum quenching on biomass accumulation: A clogging control strategy in gas biofilters. *Chemical Engineering Journal* **2022,** *432*, 134313.

15. Skorupski, K.; Taylor, R. K., A new level in the Vibrio cholerae ToxR virulence cascade: AphA is required for transcriptional activation of the tcpPH operon. *Molecular microbiology* **1999,** *31*, (3), 763-771.

16. Rutherford, S. T.; Van Kessel, J. C.; Shao, Y.; Bassler, B. L., AphA and LuxR/HapR reciprocally control quorum sensing in vibrios. *Genes & development* **2011,** *25*, (4), 397-408.

17. Sledjeski, D. D.; Whitman, C.; Zhang, A., Hfq is necessary for regulation by the untranslated RNA DsrA. *Journal of bacteriology* **2001,** *183*, (6), 1997-2005.

18. Essar, D. W.; Eberly, L.; Hadero, A.; Crawford, I., Identification and characterization of genes for a second anthranilate synthase in Pseudomonas aeruginosa: interchangeability of the two anthranilate synthases and evolutionary implications. *Journal of bacteriology* **1990,** *172*, (2), 884-900.

19. Bever, R. A.; Iglewski, B. H., Molecular characterization and nucleotide sequence of the Pseudomonas aeruginosa elastase structural gene. *Journal of bacteriology* **1988,** *170*, (9), 4309-4314.

20. Cahan, R.; Axelrad, I.; Safrin, M.; Ohman, D. E.; Kessler, E., A secreted aminopeptidase of Pseudomonas aeruginosa: identification, primary structure, and relationship to other aminopeptidases. *Journal of Biological Chemistry* **2001,** *276*, (47), 43645-43652.

21. Sperandio, V.; Torres, A. G.; Kaper, J. B., Quorum sensing Escherichia coli regulators B and C (QseBC): a novel two‐component regulatory system involved in the regulation of flagella and motility by quorum sensing in E. coli. *Molecular microbiology* **2002,** *43*, (3), 809-821.

22. Bartlett, D.; Frantz, B.; Matsumura, P., Flagellar transcriptional activators FlbB and FlaI: gene sequences and 5'consensus sequences of operons under FlbB and FlaI control. *Journal of bacteriology* **1988,** *170*, (4), 1575-1581.

23. Reading, N. C.; Torres, A. G.; Kendall, M. M.; Hughes, D. T.; Yamamoto, K.; Sperandio, V., A novel two-component signaling system that activates transcription of an enterohemorrhagic Escherichia coli effector involved in remodeling of host actin. *Journal of bacteriology* **2007,** *189*, (6), 2468-2476.

24. Psakis, G.; Mailliet, J.; Lang, C.; Teufel, L.; Essen, L.-O.; Hughes, J., Signaling kinetics of cyanobacterial phytochrome Cph1, a light regulated histidine kinase. *Biochemistry* **2011,** *50*, (28), 6178-6188.

25. Pacheco, A. R.; Curtis, M. M.; Ritchie, J. M.; Munera, D.; Waldor, M. K.; Moreira, C. G.; Sperandio, V., Fucose sensing regulates bacterial intestinal colonization. *Nature* **2012,** *492*, (7427), 113-117.

26. Black, P. N.; DiRusso, C. C.; Metzger, A. K.; Heimert, T. L., Cloning, sequencing, and expression of the fadD gene of Escherichia coli encoding acyl coenzyme A synthetase. *Journal of Biological Chemistry* **1992,** *267*, (35), 25513-25520.

27. Ryan, R. P.; Fouhy, Y.; Lucey, J. F.; Crossman, L. C.; Spiro, S.; He, Y.-W.; Zhang, L.-H.; Heeb, S.; Cámara, M.; Williams, P., Cell–cell signaling in Xanthomonas campestris involves an HD-GYP domain protein that functions in cyclic di-GMP turnover. *Proceedings of the National Academy of Sciences* **2006,** *103*, (17), 6712-6717.

28. Chatterjee, S.; Almeida, R. P. P.; Lindow, S., Living in two worlds: the plant and insect lifestyles of Xylella fastidiosa. *Annu. Rev. Phytopathol.* **2008,** *46*, 243-271.

29. Dow, M., Diversification of the function of cell-to-cell signaling in regulation of virulence within plant pathogenic xanthomonads. *Science signaling* **2008,** *1*, (21), pe23-pe23.

30. Tao, F.; Swarup, S.; Zhang, L. H., Quorum sensing modulation of a putative glycosyltransferase gene cluster essential for Xanthomonas campestris biofilm formation. *Environmental microbiology* **2010,** *12*, (12), 3159-3170.

31. Sadaie, Y.; Nakadate, H.; Fukui, R.; Yee, L. M.; Asai, K., Glucomannan utilization operon of Bacillus subtilis. *FEMS microbiology letters* **2008,** *279*, (1), 103-109.

32. Tao, F.; He, Y.-W.; Wu, D.-H.; Swarup, S.; Zhang, L.-H., The cyclic nucleotide monophosphate domain of Xanthomonas campestris global regulator Clp defines a new class of cyclic di-GMP effectors. *Journal of bacteriology* **2010,** *192*, (4), 1020-1029.

33. Bai, G.; McCue, L. A.; McDonough, K. A., Characterization of Mycobacterium tuberculosis Rv3676 (CRPMt), a cyclic AMP receptor protein-like DNA binding protein. *Journal of bacteriology* **2005,** *187*, (22), 7795-7804.

34. He, Y. W.; Ng, A. Y. J.; Xu, M.; Lin, K.; Wang, L. H.; Dong, Y. H.; Zhang, L. H., Xanthomonas campestris cell–cell communication involves a putative nucleotide receptor protein Clp and a hierarchical signalling network. *Molecular microbiology* **2007,** *64*, (2), 281-292.

35. Nahm, C. H.; Choi, D.-C.; Kwon, H.; Lee, S.; Lee, S. H.; Lee, K.; Choo, K.-H.; Lee, J.-K.; Lee, C.-H.; Park, P.-K., Application of quorum quenching bacteria entrapping sheets to enhance biofouling control in a membrane bioreactor with a hollow fiber module. *Journal of Membrane Science* **2017,** *526*, 264-271.

36. Ergön-Can, T.; Köse-Mutlu, B.; Koyuncu, İ.; Lee, C.-H., Biofouling control based on bacterial quorum quenching with a new application: Rotary microbial carrier frame. *Journal of Membrane Science* **2017,** *525*, 116-124.

37. Lee, S. H.; Lee, S.; Lee, K.; Nahm, C. H.; Kwon, H.; Oh, H.-S.; Won, Y.-J.; Choo, K.-H.; Lee, C.-H.; Park, P.-K., More efficient media design for enhanced biofouling control in a membrane bioreactor: quorum quenching bacteria entrapping hollow cylinder. *Environmental science & technology* **2016,** *50*, (16), 8596-8604.

38. Weerasekara, N. A.; Choo, K.-H.; Lee, C.-H., Hybridization of physical cleaning and quorum quenching to minimize membrane biofouling and energy consumption in a membrane bioreactor. *Water research* **2014,** *67*, 1-10.

39. Kim, S.-R.; Oh, H.-S.; Jo, S.-J.; Yeon, K.-M.; Lee, C.-H.; Lim, D.-J.; Lee, C.-H.; Lee, J.-K., Biofouling control with bead-entrapped quorum quenching bacteria in membrane bioreactors: physical and biological effects. *Environmental science & technology* **2013,** *47*, (2), 836-842.

40. Oh, H.-S.; Yeon, K.-M.; Yang, C.-S.; Kim, S.-R.; Lee, C.-H.; Park, S. Y.; Han, J. Y.; Lee, J.-K., Control of membrane biofouling in MBR for wastewater treatment by quorum quenching bacteria encapsulated in microporous membrane. *Environmental science & technology* **2012,** *46*, (9), 4877-4884.

41. Oh, H.-S.; Kim, S.-R.; Cheong, W.-S.; Lee, C.-H.; Lee, J.-K., Biofouling inhibition in MBR by Rhodococcus sp. BH4 isolated from real MBR plant. *Applied microbiology and biotechnology* **2013,** *97*, (23), 10223-10231.
